# Statistical and machine learning methods for spatially resolved transcriptomics data analysis

**DOI:** 10.1186/s13059-022-02653-7

**Published:** 2022-03-25

**Authors:** Zexian Zeng, Yawei Li, Yiming Li, Yuan Luo

**Affiliations:** 1 Center for Quantitative Biology, Academy for Advanced Interdisciplinary Studies, Peking University, Beijing, 100084 China; 2 Peking-Tsinghua Center for Life Sciences, Academy for Advanced Interdisciplinary Studies, Peking University, Beijing, 100084 China; 3 Department of Data Sciences, Dana Farber Cancer Institute, Harvard T.H. Chan School of Public Health, Boston, MA 02215 USA; 4 Division of Health and Biomedical Informatics, Department of Preventive Medicine, Northwestern University Feinberg School of Medicine, Chicago, IL 60611 USA; 5 Northwestern University Clinical and Translational Sciences Institute, Chicago, IL 60611 USA; 6 Institute for Augmented Intelligence in Medicine, Northwestern University, Chicago, IL 60611 USA; 7 Center for Health Information Partnerships, Northwestern University, Chicago, IL 60611 USA

## Abstract

The recent advancement in spatial transcriptomics technology has enabled multiplexed profiling of cellular transcriptomes and spatial locations. As the capacity and efficiency of the experimental technologies continue to improve, there is an emerging need for the development of analytical approaches. Furthermore, with the continuous evolution of sequencing protocols, the underlying assumptions of current analytical methods need to be re-evaluated and adjusted to harness the increasing data complexity. To motivate and aid future model development, we herein review the recent development of statistical and machine learning methods in spatial transcriptomics, summarize useful resources, and highlight the challenges and opportunities ahead.

**Supplementary Information:**

The online version contains supplementary material available at 10.1186/s13059-022-02653-7.

## Introduction

**Fig. 1:**
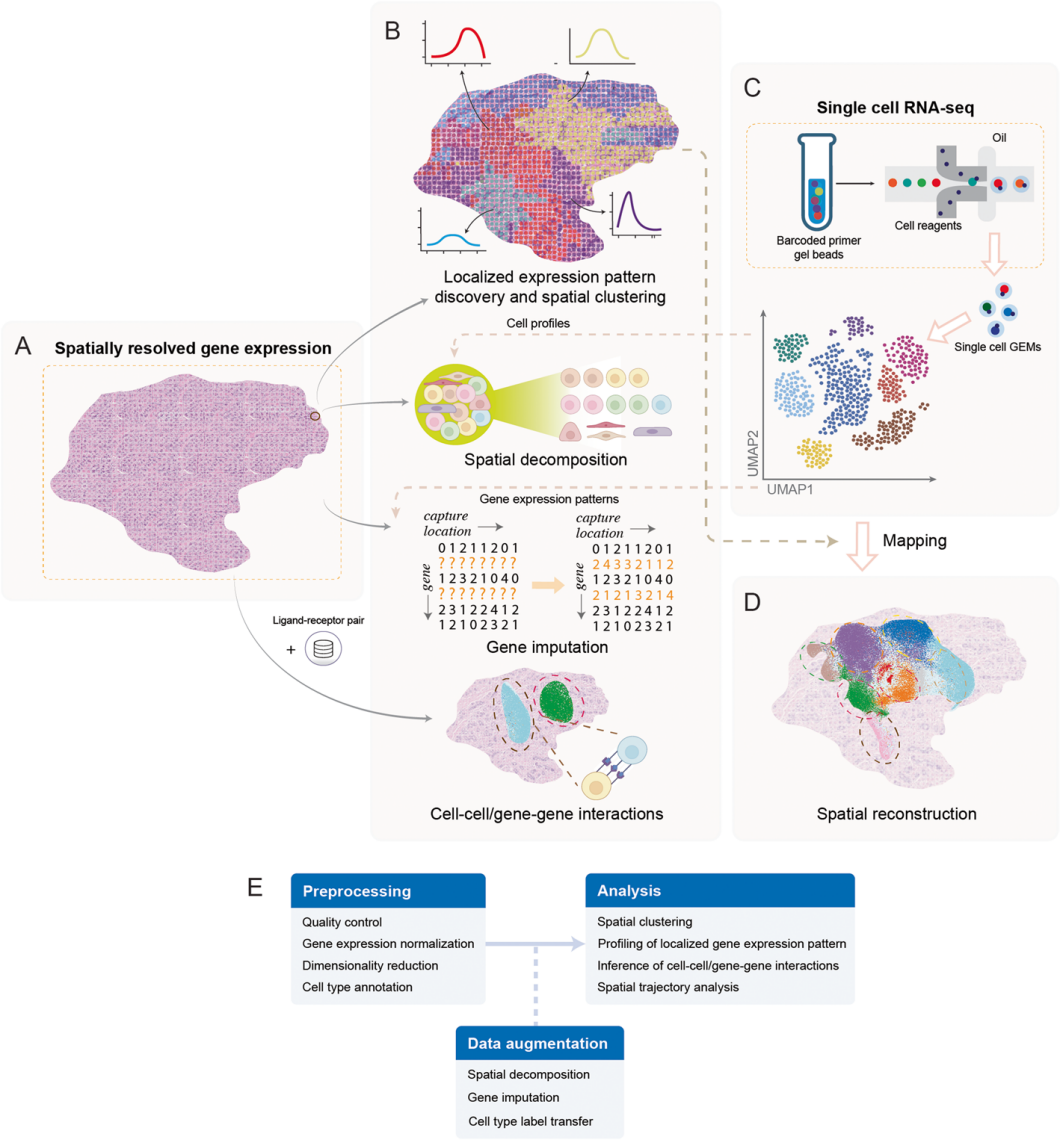
Applications of computational approaches in spatial transcriptomics research. **A** Spatially resolved transcriptomics measures transcriptomes while preserving spatial information. Although spatial transcriptomics data retains spatial information, it is compromised with low cellular resolution and read coverage. **B** Computational approaches capable of harnessing the complexity of spatial transcriptomics data have been developed for applications of localized gene expression pattern identification, spatial decomposition, gene imputation, and cell-cell interaction. Some of these models leverage gene expression profiles from single-cell RNA-seq (scRNA-seq) data or prior ligand-receptor information from relevant databases to aid spatial transcriptomics research. **C** Sequencing protocols for scRNA-seq have achieved high-throughput profiling at single-cell resolution, but cellular spatial information is lost during sequencing. Compared to spatial transcriptomics, scRNA-seq is more accessible and can reach cellular resolution. **D** By leveraging information from the spatial transcriptomics data, spatial location reconstruction could be performed for scRNA-seq data with missing spatial information. In addition, spatial locations could be reconstructed de novo by integrating prior knowledge such as ligand-receptor pair information. **E** A typical analysis workflow for spatial transcriptomics data. GEMs, Gel beads-in-emulsions; UMAP, uniform manifold approximation and projection

In unicellular and multicellular organisms, arranged cells work collaboratively in intact tissues. Spatially resolved transcriptomics performs high-throughput measurement of transcriptomes while preserving spatial information about the tissue context and cellular organizations [1–8] [spatial transcriptomics technologies were reviewed in [9–12]] (Fig. 1A). In the past decade, the rapid development of spatial transcriptomics technology has facilitated biological discoveries in different domains [4, 13–15]. Spatially resolved transcriptomics enables us to study cell transcriptomes in the context of cellular organizations. This additional dimension of spatial information has shown its efficacies in providing us with a novel perspective on the cellular transcriptome [successful applications of spatial transcriptomics for biological discoveries were reviewed in [16, 17]]. Meanwhile, advances in spatial transcriptomics have increased the data volume and complexity and introduced new challenges for data analysis (Fig. 1B). The recent development of computational approaches has created new effective paradigms for analyzing high-dimensional data, e.g., in single-cell RNA-seq (scRNA-seq) research [18]. Likewise, there has been much progress in the field of method development for spatial transcriptomics data analysis [19, 20]. Theoretically, many of the computational approaches developed for scRNA-seq data analysis could be adapted to study spatial transcriptomics data. Nevertheless, designing new approaches is still necessary to take full advantage of the spatial information.

ScRNA-seq and spatial transcriptomics data are complementary to each other, and each has its unique properties and strengths. Protocols for scRNA-seq have achieved high-throughput gene expression profiling. Although information about the cellular spatial location is lost during cell preparation (Fig. 1C), the lost information has the potential to be reconstructed by leveraging the gene expression patterns of the cells. On the other hand, spatial transcriptomics retains spatial information, but majority of the data is neither transcriptome-wide in breadth nor at cellular resolution in depth. For example, when a sequencing capture location is larger than an individual cell, gene expressions measured at that capture location will be from a mixture of multiple cells. To solve this problem, we may adapt the idea of transfer learning, which utilizes knowledge learned from similar domains where data is more accessible or better labeled [21]. Indeed, by leveraging both expression profiles from scRNA-seq data and spatial patterns from spatial transcriptomics data, we can transfer knowledge between the two types of data, which benefits the analysis of both data types. It has been shown that the integration of scRNA-seq and spatial transcriptomics data could improve model performance in different research areas, including cell type annotation, cell clustering, spatial decomposition, gene imputation, cell label transfer, and spatial location reconstruction (Fig. 1B, D) [the benefits of integrating scRNA-seq and spatial transcriptomics data were reviewed in [22]].

The main objective of this review is to dissect different problems motivating method development for spatial transcriptomics, highlight their current solutions, and specify the underlying assumptions. A spatial transcriptomics data analysis workflow typically includes multiple phases (Fig. 1E). The first step is data preprocessing, which usually includes quality control, gene expression normalization, dimension reduction, and cell type annotation. One may further improve the data enrichment with spatial decomposition, gene imputation, and label transfer. Next, one could gain biological insights from the data through spatial clustering and localized gene expression pattern discovery, which could further facilitate the identification of spatially variable genes, inference of cell-cell/gene-gene interactions, and spatial trajectory analysis. Furthermore, spatial transcriptomics data can be utilized to help reconstruct spatial locations in the scRNA-seq data (Fig. 1E). Despite the current successful applications of computational methods in this workflow, there still exists an urgent need to develop more sophisticated models to tackle the rising challenges in spatial transcriptomics data analysis. To bridge the gap between evolving experimental technology and current computational techniques, we herein survey the applications of computational methods in spatial transcriptomics and classify them into major categories based on the domain of applications (Table 1). We begin with the analytical approaches characterizing localized gene expression patterns and performing spatial clustering. We also discuss strategies for improving the data enrichment, including spatial decomposition and gene imputation (Fig. 1B). Next, we review computational methods that learn patterns from spatial transcriptomics data to help reconstruct spatial information for scRNA-seq data (Fig. 1D). Lastly, we focus on the computational methods that leverage spatial transcriptomics data to aid cell-cell communication and gene-gene interaction inference (Fig. 1B). We conclude by outlining the challenges and future opportunities in the field of method development for spatial transcriptomics. We also summarize useful datasets (Additional file 1: Table S1), baseline methods for benchmark studies (Additional file 1: Table S2), and available data processing pipelines to assist further investigations. We anticipate that this review will motivate future method development to address the increasing complexity of spatial transcriptomics data.

**Table 1:** A summary of algorithms, application scenarios, advantages, and disadvantages of the reviewed methods

Name	Algorithms	Application scenarios	Advantages	Disadvantages
SpatialDWLS [23]	Weighted least squares	Spatial decomposition	Higher accuracy and faster than benchmarked tools	High bias in estimating the proportion of rare cell types
SPOTlight [24]	Seeded NMF regression	Spatial decomposition	High accuracy across multiple tissues	Does not incorporate capture location information to model spatial decomposition
RCTD [25]	Poisson distribution with MLE	Spatial decomposition	Systematically models platform effect	Assumes that platform effects are shared among cell types
*stereoscope* [26]	Negative binomial distribution with MAP	Spatial decomposition	Utilizes complete expression profiles rather than selected marker genes to achieve a higher accuracy	Requires deep sequencing depth
DSTG [27]	Semi-supervised GCN	Spatial decomposition	Higher accuracy than benchmarked tools	Highly dependent on the quality of the link graph that models the GCN
ProximID [28]	Cluster label permutations	Cell-cell/gene-gene interactions	Does not require to physically separate the cells in FISH images	Cannot detect interactions that are not physically attached
MISTy [29]	Multi-view framework to dissect effects related to CCI	Cell-cell/gene-gene interactions	1. Does not require cell type annotation 2. Utilizes complete expression profiles	The extracted interactions cannot be directly considered as causal
stLearn [30]	A toolbox containing integrated algorithms from multiple studies	1.Cell-cell/gene-gene interactions 2. Spatial clustering 3. Cell trajectories inference	A streamlined package from raw inputs to in-depth downstream analysis	Only compatible with certain ST platforms
SVCA [31]	Gaussian processes	Cell-cell/gene-gene interactions	Is applicable to both RNA-seq and proteomic data	Does not account for technology-specific noise
GCNG [32]	GCN	Cell-cell/gene-gene interactions	Can infer novel CCIs and predict novel functional genes	The hyperparameters need to be re-optimized when applied to different datasets
Seurat V3 [33]	Analysis pipelines with integrated algorithms	1. Gene imputation 2. Spatial location reconstruction for scRNA-seq data 3. Others	1. A comprehensive data analysis pipeline 2. Can be applied to multi-omics datasets, including transcriptomic, epigenomic, proteomic, and spatially resolved single-cell data	Only available for certain types of ST platforms
LIGER [34]	Integrative NMF	1. Gene imputation 2. Spatial location reconstruction for scRNA-seq data	The embeddings maintain both common and dataset-specific terms	Memory intensive compared to benchmarked tools
SpaGE [35]	Domain adaptation model to align ST and scRNA-seq data to a common space	1. Gene imputation 2. Spatial location reconstruction for scRNA-seq data	Less memory usage and faster than benchmarked tools in large datasets	Only common genes in both datasets are included in the model
stPlus [36]	Autoencoder model for dimensional reduction to map ST and scRNA-seq data into a shared space	Gene imputation	1. Higher accuracy than benchmarked tools in cell type clustering 2. Less time and memory usage than most benchmarked tools other than SpaGE [35] when applied to large datasets	Only applicable to data from image-based sequencing platforms
gimVI [37]	Variational autoencoders for dimensional reduction to map ST and scRNA-seq data into a shared space	1. Gene imputation 2. Dimensional reduction and feature extraction	Generates platform-specific patterns in the model for better biological interpretability	Slower than benchmarked tools in large datasets
Harmony [38]	Maximum diversity clustering and mixture model based batch correction	1. Gene imputation 2. Spatial location reconstruction for scRNA-seq data	Can impute low abundant genes with high accuracy	The embeddings lack biological interpretability
DEEPsc [39]	ANN	Gene imputation	A system-adaptive method specifically designed for gene imputation	Does not incorporate spatial information into the computation
Trendsceek [40]	Marked point process	Identify SVGs	Does not need to specify a distribution or a spatial region of interest	Limited to a single gene at a time, computationally intensive
SpatialDE [41]	Gaussian process regression	Identify SVGs	Can detect both temporal and periodic gene expression patterns for SVG identification	Does not identify spatial regions with distinct expression patterns, computationally intensive
SPARK [42]	Generalized linear spatial models	1. Identify SVGs 2. Spatial location reconstruction for scRNA-seq data	1. Low false discovery rate 2. Does not require the user to preprocess the raw count matrix	The hyperparameters (kernels and weights) need to be re-optimized when applied to different datasets
SpaGCN [43]	GCN	1. Identify SVGs 2. Spatial location reconstruction for scRNA-seq data	Jointly identifies SVGs and spatial domains	Does not incorporate cell type information and tissue anatomical structure into the computation
SPARK-X [44]	Non-parametric covariance test	1. Identify SVGs 2. Spatial location reconstruction for scRNA-seq data	Less time and memory usage and lower false discovery rate than most benchmarked tools, especially in large-scale and sparse ST data	Accuracy varies on different similarity measurements and covariance functions
*sepal* [45]	Diffusion model	1. Identify SVGs 2. Spatial location reconstruction for scRNA-seq data	Can detect genes with irregular spatial patterns	Has CPU parallelization, but no GPU acceleration
GLISS [46]	Graph Laplacian-based model	1. Identify SVGs 2. Spatial location reconstruction for scRNA-seq data	Does not need to make distributional assumptions for either spatial or scRNA-seq data	Requires pre-specified landmark genes either manually or through other algorithms
Zhu et al. [47]	HMRF	1. Profile localized gene expression pattern 2. Identify SVGs 3. Identify interactions between cell type and spatial environment	Can identify de novo spatially associated subpopulations	Only available for in situ hybridization datasets
BayesSpace [48]	Bayesian statistical method	1. Profile localized gene expression pattern to enhance ST data resolution 2. Spatial clustering	Does not require independent single-cell data	Only considers the neighborhood structure present in data from ST and Visium platforms
Bergenstråhle et al. [49]	Deep generative model	Gene expression prediction from histology images	Available for gene expression inference at transcriptome-wide level in histology images	Only in situ RNA capturing technologies are available
Seurat V1 [50]	L1-constrained linear model	1. Spatial location reconstruction for scRNA-seq data 2. Gene imputation	The idea of landmark genes allows the use of a small number of genes for spatial location reconstruction	Need to pre-compute the positions of landmark genes
CSOmap [51]	Reconstructs cellular spatial organization based on cell-cell affinity by ligand-receptor interactions	1. Identify cell-cell/gene-gene interactions 2. Spatial location reconstruction for scRNA-seq data	Does not need to predefine the tissue shape for cell-cell interaction inference Does not need to pre-define landmark gene sets	The extracted spatial structure is a pseudo-space structure
DistMap [52]	Mapping scores to measure the similarity between spatial and scRNA-seq data	Construct 3D gene expression blueprint for the Drosophila embryo	High accuracy with only 84 in situ suffices	Gene regulation can be considered as the in situ suffices to improve the accuracy of model
Peng et al. [53]	Spearman rank correlation to measure the similarity between spatial and scRNA-seq data	Spatial location reconstruction for scRNA-seq data	High accuracy with a small number of genes and cells required	No benchmark studies for accuracy comparison
Achim et al. [54]	Measure correlations between spatial and scRNA-seq data	Spatial location reconstruction for scRNA-seq data	Most cells can be mapped with high confidence with only a small number of marker genes (~ 50 to 100)	Need to filter low-quality genes before modeling
SpaOTsc [55]	Structured optimal transport model	1. Spatial location reconstruction for scRNA-seq data 2. Cell-cell/gene-gene interactions 3. Identify gene pairs that potentially intercellularly regulate each other	1. Most cells can be accurately mapped with only a small number of genes 2. Can identify intercellular gene-gene regulatory information	Does not consider the time delay (including the diffusion time of ligand or the reacting time of the intracellular cascades) that may take place in cell-cell communication
novoSpaRc [56]	Generalized optimal-transport model	Spatial location reconstruction for scRNA-seq data	Does not need to specify landmark genes for alignment	The accuracy can be promoted by using different loss functions
Tangram [57]	Non-convex optimization by deep learning methods for spatial alignment	1. Spatial location reconstruction for scRNA-seq data 2. Spatial decomposition 3. Gene imputation from histology data	Is compatible with both capture-based and image-based ST data	Histology gene expression prediction is less accurate if cells cannot be segmented in the images
Cell2location [58]	Hierarchical Bayesian framework	1. Spatial location reconstruction for scRNA-seq data 2. Spatial decomposition	Capable of inferring the absolute number of cells per cell type for each capture location	Hyperparameters to be pre-specified are often unknown by the user
SC-MEB [59]	HMRF based on empirical Bayes	Spatially clustering	Faster and more accurate than benchmarked tools, especially in large datasets	The assumption of a fixed hexagonal neighborhood structure in the model may not maintain high accuracy for all ST platforms
STAGATE [60]	Graph attention auto-encoder	1. Spatially clustering 2. Identify SVGs	Can be applied to three-dimensional ST datasets	The boundary of two sections needs to be further refined
MULTILAYER [61]	Agglomerative clustering of quantile normalized ST data	1. Spatially clustering 2. Identify SVGs	Higher accuracy than benchmarked tools when applied to data from different ST platforms	Sensitive to ST data with low spatial resolution
HisToGene [62]	Attention-based (vision transformer) model	Gene expression prediction from histology images	Can predict the gene expression in histology images at capture location level	Requires a large number of tissue samples for model training
STARCH [63]	HMRF and HMM	Infer copy number aberrations	Higher accuracy than benchmarked tools in predicting CNAs in spatial datasets	A limited number of CNV states (deletion, neutral, amplification) are considered
Giotto [64]	A toolbox containing integrated algorithms from multiple studies	A comprehensive toolbox for ST analysis and visualization	Offers comprehensive pipelines for ST data analysis	Only available for some ST platforms

*Abbreviations*: *MLE* maximum-likelihood estimation, *MAP* maximum a posteriori, *GCN* graph convolutional network, *GNN* graph neural network, *NMF* non-negative matrix factorization, *PCA* principal components analysis, *HMRF* hidden Markov random field, *ANN* artificial neural network, *MCC* Matthews correlation coefficient, *HMM* hidden Markov model, *SVG* spatially variable gene, *CNA* copy number alteration, *CNV* copy number variation, *ST* spatial transcriptomics, *CCI* cell-cell interaction, *FISH* fluorescence in situ hybridization

## Profiling of localized gene expression pattern

**Fig. 2:**
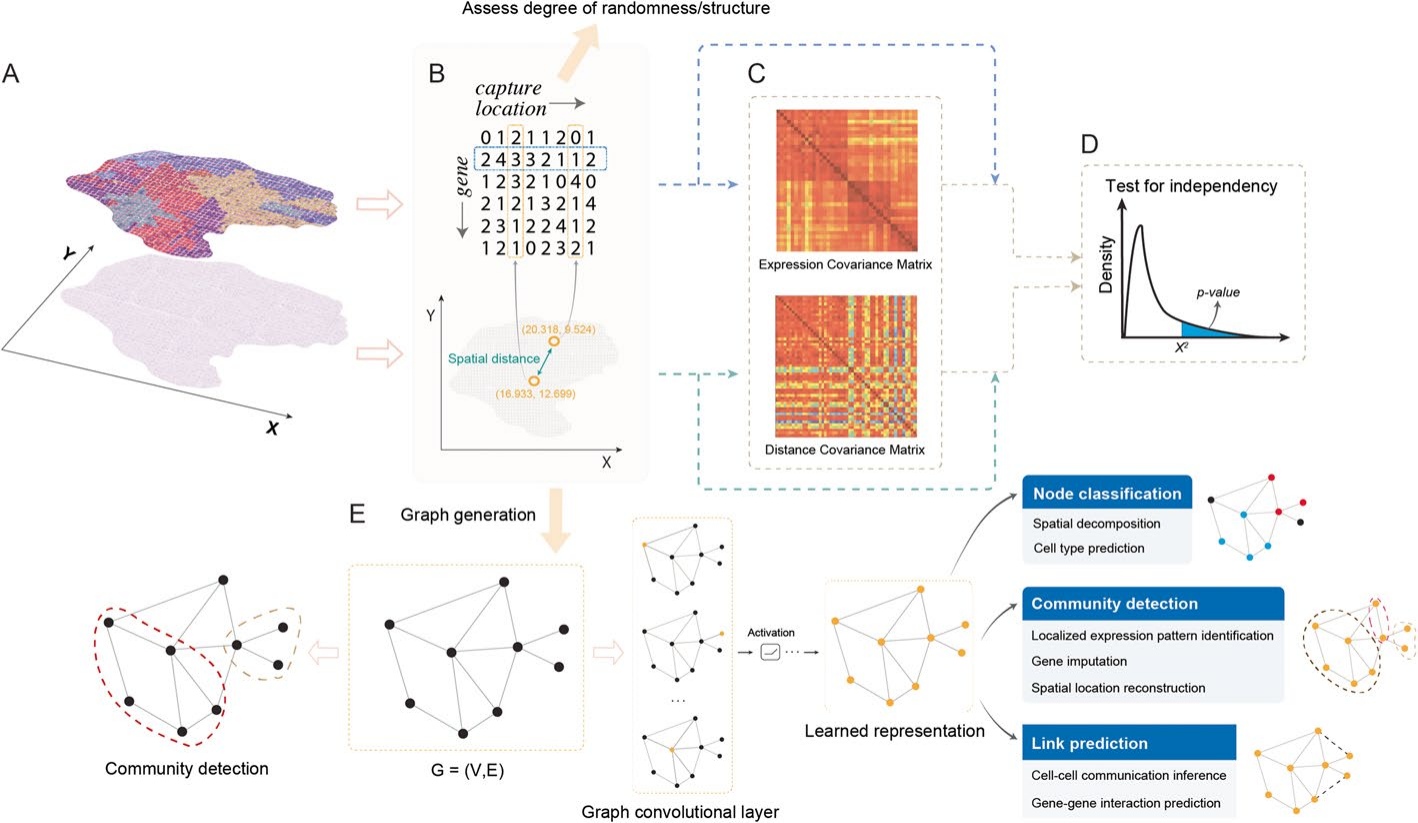
Model workflow testing independencies between gene expression and spatial locations in spatial transcriptomics data. **A** Spatial transcriptomics technology has enabled multiplexed profiling of cellular transcriptomes and spatial locations. **B** In spatial transcriptomics data, the transcriptome information is represented by a matrix with genes as rows and spatial locations as columns. Distances between the spatial locations are obtained based on their coordinates. **C** Covariance matrices of gene expressions and spatial coordinates are calculated based on the gene expression and spatial coordinates, respectively. **D** Test of significance on whether the gene expressions are independent of the spatial coordinates using the covariance matrices. **E** Model spatial transcriptomics data using graphs, where each node corresponds to a spatial location, and two nodes are connected if they have proximate locations or similar expression profiles. Graph convolutional networks can aggregate features from each spatial location’s neighbors through convolutional layers and utilize the learned representation to perform node classification, community detection, and link prediction. Extended applications include spatial decomposition, localized expression pattern identification, and cell-cell interaction inference

Genes differentially expressed with varying spatial patterns reflect biological functions. Early approaches of localized gene expression pattern identification include Trendsceek [40] and SpatialDE [41]. Trendsceek [40] utilizes the marked point process theory [65], in which spatial locations are represented as points and expression levels as marks. For a given gene, Trendsceek [40] tests whether the distributions of the gene expression (mark) are conditionally dependent on the spatial location (point). The significance of the dependency is assessed through a resampling procedure, during which gene expressions are permutated between spatial locations to generate the null distribution. For a given gene, SpatialDE [41] utilizes Gaussian process regression to decompose the expression variation into a spatial component and a non-spatial component. Specifically, the spatial component of the expression variation is modeled by the spatial covariance matrix based on the pairwise spatial distances among locations (Fig. 2A–C), and the non-spatial component is formulated as a noise term. To perform significance testing, SpatialDE [41] compares the likelihood of its full model with the likelihood of a null model without the spatial component. Similar to SpatialDE [41], SPARK [42] is a generative model with a variety of kernels to detect genes with spatial variation. A Poisson link is used in the generalized linear model as the authors reasoned that spatial gene expression data is often present in the form of counts. In addition, SPARK [42] computes *p*-values using each of the kernels and utilizes the Cauchy combination rule [66] to combine the *p*-values. Using this approach, SPARK [42] produces well-calibrated *p*-values to control type I errors. A common drawback for Trendsceek [40], SpatialDE [41], and SPARK [42] is their high computational complexity, which hinders these methods from being readily applicable to large-scale high-throughput spatial transcriptomics data. Although SpatialDE [41] and SPARK [42] are more efficient than Trendsceek [40], the computational complexity of these two methods [41, 42] still scales cubically with respect to the number of spatial locations. To reduce computational burden, SPARK-X [44] proposes a scalable non-parametric model using the following algebraic manipulations. For a given gene, SPARK-X [44] first builds a covariance matrix for the gene expression and a covariance matrix for the spatial coordinates (Fig. 2C). Intuitively, if the gene expressions are independent of the spatial coordinates, the product of the two covariance matrices will be small. Conversely, if the gene expressions are not independent of the spatial coordinates, the product of the two matrices will be large. This product is assumed to follow a mixture of chi-square distributions which allows for significance testing (Fig. 2D). A common theme of these approaches [40–42, 44] is that they all test whether adding a spatial component to the covariance could significantly improve their ability to identify spatially variable genes. We noted that SpatialDE2 [67] unifies the mapping of tissue zones and spatial variable gene detection as integrated framework.

With the common goal of identifying spatially variable genes, multiple machine learning algorithms have been proposed to examine the spatial transcriptomics data from different angles. *sepal* [45] explores the alternative solutions to hypothesis testing and assesses the degree of randomness exhibited by the data. Specifically, *sepal* [45] simulates diffusions of the gene expressions in the spatial domain and models the expression diffusion with Fick’s second law to measure the time of convergence. In this context, *sepal* [45] assumes that genes with spatial patterns will demonstrate a lower degree of randomness (diffusions) and a higher degree of structure. Therefore, compared to genes with a uniform pattern across different spatial locations, transcripts following structured patterns require more iterations for the gradient algorithm to converge [45], and a long convergence time of the system is indicative of a structured spatial pattern. On a separate note, graph-based methods have shown their efficacies in studying spatial variable genes as they could, for each node, aggregate information from its neighbors. SpaGCN [68] is a graph convolutional network (GCN) approach that integrates gene expression data, spatial location information, and histology images to identify genes with spatial patterns. The core of GCN is its graph convolutional layer, which enables it to combine graph structure (cell location and neighborhood) and node information (gene expression in the specific cell) as inputs to a convolutional network. When applied to spatial transcriptomics data, GCN could aggregate feature information from each cell/location’s adjacent cells/locations through the convolutional layers and improve model performance. In SpaGCN [68], the spatial locations are used as nodes in the input graph and are connected via edges weighted by the relatedness between different locations. The relatedness is measured by physical distance and histology similarity between spatial locations. Specifically, the histology relatedness is measured based on pixel color similarities. Through graph convolution and clustering, SpaGCN [68] next aggregates expression and relatedness information and clusters the spatial locations into domains based on the aggregated profiles. GLISS [46] is another graph-based method that constructs a mutual nearest neighborhood graph from spatial coordinates and relies on a graph-based feature selection procedure to select spatially variable genes. Similar to SpaGCN [68], GLISS [46] constructs a neighborhood graph from the spatial coordinates, with each node representing a spatial location. In the graph, two nodes will be connected if they are spatially proximate to each other. Specifically, GLISS [46] calculates the graph Laplacian score to measure the relationship between the gene expression and the constructed graph. The graph Laplacian scores are always non-negative, and small scores indicate strong dependencies of the gene expression on the spatial coordinates. Lastly, GLISS [46] performs significance testing through permutations, in which gene expression is randomly shuffled to generate the null distribution. To summarize, in the proposed graph-based models [46, 47, 68], it has been observed that aggregating feature information from each node’s neighbors improves the identification of localized gene expression patterns and, consequently, spatially variable genes.

## Spatial clustering

The profiling of localized gene expression patterns is closely related to delineating spatially connected regions or clusters in a tissue based on expression data [69]. Indeed, spatial clustering is a critical step when performing exploratory analysis of spatial transcriptomics data, which may help reduce the data dimensionality and discover spatially variable genes. Standard clustering methods designed for scRNA-seq data were often based on gene expression levels, whereas spatial clustering requires us to take spatial information into account. To profile localized gene expression patterns, stLearn [30] first normalizes the expression data by smoothing the expression values in each capture location upon information aggregated from its neighbors and is weighted by the morphological similarity between capture locations. The capture locations are next clustered using standard algorithms such as *k*-means, and spatial information is used to refine the cluster results by merging subclusters from expression-driven clusters that are split across multiple spatially separated locations [30]. Inspired by contextual image classification methods, MULTILAYER [61] evaluates each gene’s differential expression level by comparing it to the average expression in the whole tissue and applies hierarchical agglomerative clustering to identify the gene expression patterns. These patterns are represented by nodes in a graph, in which the edges are weighted by the similarities of gene patterns [61]. Tissue communities are next detected by applying Louvain clustering to the graph [61]. There are also examples that utilize Markov random fields (MRF) to incorporate spatial information when performing spatial clustering. Zhu et al. [47] utilize hidden Markov random fields (HMRF) to identify spatially variable domains. The authors first construct a neighborhood graph to represent the spatial relationship among the capture locations. In this work, the cell states depend on the label of their immediate neighbor nodes. The model also incorporates a term to compare the expression of a cell to the different clusters. Therefore, the HMRF model forces the clusters to be coherent both in physical and gene expression space. The authors leverage HMRF to decompose the graph into multiple components, and each component represents a spatially variable domain [47]. In another work, BayesSpace [48] employs a Bayesian approach and impose a prior to assign higher weights to spatial locations that are physically closer. However, the performance of BayesSpace [48] might be limited by its fixed smoothing parameter of the MRF. Moreover, it is not computationally scalable for high-throughput spatial transcriptomics data since the Markov chain Monte Carlo (MCMC) part is computationally intensive [48]. To address these drawbacks, SC-MEB performs spatial clustering through an empirical Bayes approach capable of optimizing the smoothness parameter [59]. The gene expression at each capture location is assumed to be Gaussian given an unknown cluster label, and the prior of the hidden labels encourages spatial smoothness by penalizing the assignment of neighboring capture locations to different clusters [59]. SC-MEB estimates its parameters using an iterative-conditional-mode-based expectation-maximization method to boost its computational efficiency and scalability to high-throughput data [59]. Another strategy for spatial clustering is to perform graph-based clustering using both gene expression profiles and spatial features. For example, STAGATE [60] is a graph attention auto-encoder framework capable of identifying spatial clusters. It first constructs a neighborhood graph of the capture locations and prunes the graph based on the clustering of gene expressions [60]. The similarity between neighboring capture locations in the spatial graph is estimated by an attention layer, and clustering results on the inferred latent embeddings can then provide us with informative spatial domains [60]. In addition, as discussed in the previous section, SpaGCN is a GCN-based method capable of integrating gene expression, histology images, and spatial coordinate data [68]. In SpaGCN, spatial clusters are identified through clustering the output of the graph convolutional layer [68].

## Spatial decomposition and gene imputation

**Fig. 3:**
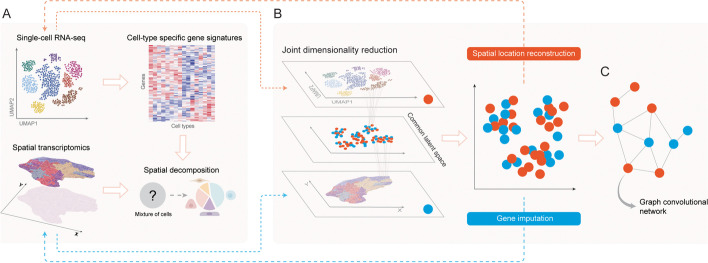
Leveraging expression profiles from scRNA-seq data and spatial patterns from spatial transcriptomics data benefits the analysis of both types of data. **A** In sequencing protocols where the size of the capture location is larger than a cell, multiple cells are profiled as a mixture. Cell type-specific expression profiles derived from scRNA-seq data can be used to estimate cell type proportions at different capture locations. **B** With both scRNA-seq and spatial transcriptomics data projected to and clustered in a common latent space, complementary information from one type of data can be used for imputing features missing from the other type, for instance, spatial pattern prediction for scRNA-seq data and gene imputation for spatial transcriptomics data. **C** Graphs can next be constructed based on the feature similarities in the latent space, allowing downstream graph-based methods such as graph convolutional networks. UMAP, uniform manifold approximation and projection

When a capture location is larger than an individual cell, its measured gene expressions may be from a mixture of multiple cell types as the capture location overlaps with multiple cells. For example, the capture locations of Visium, a widely used microarray-based spatial transcriptomics technique, are ~ 55 μm in diameter. This is often larger than a typical cell size (around 5–10 μm). Therefore, an important preprocessing step is to estimate the proportions of different cell types in each capture location using spatial decomposition algorithms, which is similar to the concept of cellular deconvolution. Traditionally, cellular deconvolution commonly refers to estimating the proportions of different cell types in each sample based on its bulk RNA-seq data. Theoretically, methods designed for bulk RNA-seq data deconvolution could be adopted for spatial transcriptomics data [methods for bulk RNA-seq deconvolution are benchmarked in [70, 71]]. DWLS [72] is a tool developed for bulk RNA-seq data deconvolution. As an extension of DWLS [72], spatialDWLS [23] was proposed for spatial transcriptomics data decomposition. Leveraging cell type signatures derived from scRNA-seq data, spatialDWLS [23] performs gene signature enrichment to infer cell types that are likely to be present at each spatial capture location. Next, spatialDWLS [23] utilizes a weighted least squares approach to infer cell type composition in each spatial location using the derived signatures (Fig. 3A). To analyze Slide-seq data, Rodriques et al. [6] propose to utilize non-negative matrix factorization (NMF) to derive metagenes from the scRNA-seq data. With gene signatures representing each cell type inferred, the authors further leverage non-negative least square (NNLS) regression to map scRNA-seq cell types onto Slide-seq data. Likewise, SPOTlight [24] was proposed to utilize scRNA-seq data and NMF for spatial decomposition. Using signatures derived from scRNA-seq data, SPOTlight [24] utilizes NNLS to decompose the spatial transcriptomics data and derive the coefficients for each cell type. The coefficients derived from the NNLS models represent cell type proportions since each coefficient corresponds to a specific cell type. It is worth noting that spatialDWLS, NNLS, and SPOTlight all use the non-negative least square regression or its variants to deconvolute the spots of spatially resolved transcriptomics data, and a major difference between them is the strategy of constructing the gene signature matrix. Another spatial decomposition method, RCTD [25], leverages cell type profiles learned from scRNA-seq data to decompose cell mixtures for spatial transcriptomics data. RCTD [25] first derives gene expression profiles for each cell type from the scRNA-seq data. For a given capture location, its total transcript count is the summation of transcripts from multiple cells. Using transcript counts as the output and each cell type’s expression profiles as input variables, RCTD [25] infers cell type proportions using maximum-likelihood estimation. *stereoscope* [26] also utilizes expression profiles from scRNA-seq data and estimates cell type proportions probabilistically (Fig. 3A). Specifically, *stereoscope* [26] assumes that spatial gene counts follow negative binomial distribution.

A more recent approach, DSTG [27], utilizes a semi-supervised GCN to decompose cell mixtures in spatial transcriptomics data. DSTG [27] first performs canonical correlation analysis (CCA) to project both scRNA-seq data and spatial transcriptomics data to a common latent space, and performs *k*-nearest neighbor (KNN) to identify mutual nearest neighbors and to construct a link graph. In the constructed link graph, two nodes are connected if they are mutually nearest neighbors (Fig. 3B). Since the cell types in scRNA-seq data are already known, this problem can be formulated as a semi-supervised learning problem, in which DSTG [27] predicts unknown cell proportions for each capture location. Other approaches have been proposed for spatial decomposition, for example, a recent method named Tangram [57]. Tangram [57] is an optimization-based approach to align scRNA-seq data onto different spatial transcriptomics data by enforcing the similarity between the two data types. It is worth noting that Tangram [57] is compatible with capture-based and image-based spatial transcriptomics data. A recent approach, Cell2location [58], is a hierarchical Bayesian model that maps the spatial distribution of cell types by leveraging information from scRNA-seq data. Cell2location [58] was systematically evaluated against other alternative methods, including *stereoscope* [26], Seurat [33], RCTD [25], NNLS (Autogenes) [73], and SPOTlight [24]. It was reported that Cell2location [58] outperformed these methods substantially in detecting the presence of cell types across locations. In sum, current spatial decomposition methods [23–26] aim to learn cell type-specific marker genes or gene signature representations from scRNA-seq data. With the derived cell type signatures, the probability of cell type mixtures in each capture location can be inferred through maximum likelihood estimation. Likewise, gene marker-based approaches have also been utilized to identify and map cell subpopulations across tissue regions [74]. It is also worth noting that sequencing protocols and efficiency are different across scRNA-seq and spatial transcriptomics platforms. Therefore, data from these two platforms might have different underlying distributions. When integrating data generated by different platforms, platform effects need to be accounted for, as has been done in [25].

Gene imputation is another major task to improve the quality of spatial transcriptomics data. Some spatial transcriptomics techniques have high capturing resolution, but they only sequence a small fraction of genes out of the entire transcriptome. For example, previous versions of MERFISH may achieve single-cell resolution, but could only sequence around 1000 genes [3]. Hence, to improve the quality of spatial transcriptomics data, one could impute the missing genes when performing data preprocessing. Since information from scRNA-seq data and spatial transcriptomics data are complementary to each other, the missing spatial gene expressions could be imputed by utilizing knowledge from scRNA-seq data. Some methods developed for spatial decomposition also have the gene imputation function, for example, Tangram [57]. For gene imputation purposes, gimVI [37], a neural network model, was proposed to integrate spatial transcriptomics data and scRNA-seq data for missing gene imputation. gimVI [37] is extended from scVI [75] and based on a hierarchical Bayesian model with conditional distributions specified by deep neural networks. Specifically, this latent representation is decoded by one additional non-linear transformation to generate a posterior estimate of the distributional parameters of each gene in each cell. gimVI [37] and scVI [75] incorporate the conditional distribution to take platform effect into consideration. In terms of aggregating scRNA-seq data and spatial transcriptomics data, gimVI [37] and scVI [75] differ from other methods in their non-linearity, as many other methods are dependent on linear models. Of note, gimVI [37] reasons that distributions of gene expression are platform-specific. It assumes a zero-inflated negative binomial (ZINB) distribution for scRNA-seq measurements, a Poisson distribution for single-molecule fluorescence in situ hybridization (smFISH) [3] measurements, and a negative binomial distribution for spatially resolved transcript amplicon readout mapping (starMAP) [76] measurements. To embed scRNA-seq data generated from different batches to a common latent space, Harmony [38] projects cells to a shared embedding with reduced dimension through iterations of maximum diversity clustering and mixture-model-based linear batch correction. In one of its applications, Harmony [38] projects scRNA-seq data and spatial transcriptomics data to a common latent space (Fig. 3B). Leveraging the embeddings in the latent space, Harmony [38] utilizes KNN imputation to predict gene expressions for spatial transcriptomics data based on their nearest scRNA-seq data neighbors. Similar to Harmony [38], other tools including LIGER [34], Seurat [33], and SpaGE [35] also rely on joint dimension reduction methods to project scRNA-seq data and spatial transcriptomics data to a common latent space before performing gene imputation (Fig. 3B). Specifically, LIGER [34] utilizes NMF, Seurat [33] utilizes CCA, and SpaGE [35] uses both principal component analysis (PCA) and singular value decomposition (SVD). Different from gimVI [37], which utilizes a non-linear deep generative model, Harmony [38], LIGER [34], Seurat [33], and SpaGE [35] utilize linear models to learn embeddings. Similar to these methods [33–35, 38], stPlus [36] also aims to identify a common latent space by performing joint embedding projection with an auto-encoder and predicting spatial gene expression based on the cells’ neighboring scRNA-seq profiles after weighted KNN clustering. In summary, a common strategy for gene imputation is to embed both scRNA-seq data and spatial transcriptomics data into a common latent space for cell clustering (Fig. 3B). With the scRNA-seq cells and spatial transcriptomics locations embedded, the general process of spatial gene imputation is to integrate information from neighboring scRNA-seq cells for each of the spatial transcriptomics locations. Common latent space construction is one of the most important steps in gene imputation. As discussed in previous sections [43, 46–48], graph-based methods integrating features from neighboring cells could enhance the identification of genes with localized expression patterns. Therefore, the application of graph-based methods may improve the imputation of spatial genes (Fig. 3C).

The computational methods developed for spatial decomposition and gene imputation are largely dependent on the integration of scRNA-seq data and spatial transcriptomics data. Joint dimension reduction methods have been commonly used for this data integration purpose. A typical workflow for joint dimension reduction is to project multiple datasets to a common latent space based on feature similarities (Fig. 3B). With multiple datasets projected and clustered, complementary information from the other datasets could be used. For example, optimal transport algorithm [77] has been used in spatial transcriptomics data analyses [55, 56] as it could derive a probabilistic embedding to minimize the discrepancy between the shortest path lengths in expression data and spatial data. The integration of scRNA-seq data and spatial transcriptomics data could improve data quality in many other ways. For example, Qian et al. developed a Bayesian model to leverage scRNA-seq data to estimate the probability of assigning each read to each cell and each cell to each class for transcriptomics data [78]. This is a typical use case of cell label transfer from scRNA-seq data to spatial transcriptomics data to assist cell type annotations. As part of the analysis workflow, cell type annotation is a major task to determine the cellular composition of complex tissues and organisms. The exponential growth in the number of cells and quality of scRNA-seq has prompted the adaption and development of computational approaches to transfer cell labels from scRNA-seq data to spatial transcriptomics data. Typically, the label transfer is performed in different ways. One could first learn gene markers or gene signatures representing cell types from the scRNA-seq data, and then computationally infer the cell types for spatial transcriptomics data by enrichment studies. Alternatively, one could integrate scRNA-seq and spatial transcriptomics data and compute their similarity to perform cell type annotation for spatial transcriptomics data, as in gimVI [37], Seurat [50], Tangram [57], and others.

To further improve the quality of spatial transcriptomics data, methods have been developed to leverage other data types in addition to scRNA-seq data. For example, xFuse [49] is a deep generative model that integrates in situ RNA capturing data with histology image data to infer transcriptome-wide expression maps. The quantification of gene expression both within and between the original capture locations enhances the resolution of spatial transcriptomics data. HistoGene [62] is another deep learning model to leverage information learned from spatial transcriptomics data to predict gene expression for tissue sections where only histology images are available. HistoGene [62] outperforms other approaches that were designed to predict gene expression profiles from whole-slide images, including a multilayer perceptron-based method HE2RNA [79] and a supervised convolutional neural network-based approach ST-Net [80].

## Spatial location reconstruction for scRNA-seq data

The integration of scRNA-seq data and spatial transcriptomics data made spatial gene imputation possible. Likewise, spatial information derived from spatial transcriptomics could help reconstruct spatial information for scRNA-seq data. Researchers have proposed different computational approaches to reconstruct the spatial organization of scRNA-seq data based on information from spatial transcriptomics data. In one of the applications, an early version of Seurat (v1.0) [50] predicts cellular locations for scRNA-seq data by referring to a small set of in situ hybridization data. From the in situ hybridization images, Seurat [50] first generates a reference map with 47 genes that are characteristic of certain spatial locations. Based on this reference map, Seurat [50] projects scRNA-seq cells to spatial locations with a probabilistic score using a bimodal mixture model (Fig. 3B). Similar to Seurat [50] which uses binarized in situ hybridization data as the reference, Achim et al. [54], DistMap [52], and others [81, 82] reconstruct cellular locations for scRNA-seq data using scoring systems that measure the similarity between spatial transcriptomics and scRNA-seq data. Peng et al. [53] propose to project scRNA-seq data to spatial locations using a reference map composed of 158 spatially variable genes through Spearman rank correlation. Specifically, the 158 variable genes are obtained by analyzing a small set of anatomically defined spatial transcriptomes of the mouse epiblast. These early approaches for spatial reconstruction often start by constructing a reference map or deriving maker genes from low-throughput in situ hybridization data. With the constructed reference map or signature, the scRNA-seq data could then be projected to the maps using correlation-based methods. For example, GLISS [46] uses lists of reference genes as prior knowledge and performs spatial location imputation for scRNA-seq data. With the advent of sequencing protocols for both scRNA-seq and spatial transcriptomics, more high-throughput data are being generated. DEEPsc [39] is a neural network-based classifier to predict spatial location for scRNA-seq data using integrated scRNA-seq and spatial transcriptomics data as input. With the model trained, DEEPsc [39] could take as input the feature vector from a single cell and predict its likelihood of spatial locations.

In situations where spatial transcriptomics data are not available for particular domains or diseases, methods have been developed to reconstruct the spatial organizations de novo for scRNA-seq data with no reliance on spatial transcriptomics data. novoSpaRc [56] was proposed to infer the location distributions for scRNA-seq data. Specifically, novoSpaRc [56] formulates the question as an optimization problem using the generalized framework of optimal transport [77]. To start, novoSpaRc [56] first calculates the shortest path lengths for each pair of cells from a KNN graph, which is constructed by correlation-based distances from the scRNA-seq data. When projecting the scRNA-seq data to spatial locations, novoSpaRc [56] aims to find a probabilistic embedding that minimizes the discrepancy between the shortest path lengths in expression data and spatial data. Intuitively, if two cells are close expression-wise, they are expected to be embedded into proximate spatial locations. Notably, in the cases where a reference map is available, novoSpaRc [56] could utilize this prior knowledge by adding a penalty term to minimize the discrepancy between the expression profiles of embedded single cells and values from the reference map. SpaOTsc [55] utilizes a similar framework as novoSpaRc [56] by solving an optimal transport problem [77] and has systematically benchmarked the method with more datasets. In particular, SpaOTsc [55] changes the penalty term from entropic regulation to unbalanced transport [83] to handle the unbalanced sample size between scRNA-seq data and spatial transcriptomics data. In order to project spatial locations to scRNA-seq data without the need for spatial transcriptomics data, CSOmap [51] assumes that cells likely to interact tend to locate in close proximity and are mediated by ligand-receptor interactions. Hence, the spatial pattern could be deciphered by utilizing ligand-receptor co-expression patterns. Under this assumption, CSOmap [51] reconstructs cellular spatial locations by performing *t*-distributed stochastic neighbor embedding (t-SNE) to embed the scRNA-seq cells into a three-dimensional map based on a cell-by-cell affinity matrix learned from ligand-receptor expression networks [84].

In summary, spatial location reconstruction for scRNA-seq data is often performed in two steps—the feature engineering step to extract reference information from spatial transcriptomics data [50, 52, 54, 81, 82] and the model building step to infer spatial location probabilities of the cells in scRNA-seq data. Theoretically, methods designed for spatial gene expression pattern identification [see the “Profiling of localized gene expression pattern” section [40–46]] could be adopted to build a spatial reference map utilizing spatial transcriptomics data. It is also worth noting that Bageritz et al. [85] have a set of genes with spatially expression patterns, which can potentially be used as a spatial reference map. Additionally, methods including Harmony [38], LIGER [34], Seurat [33], and SpaGE [35] rely on joint dimension reduction to embed both scRNA-seq data and spatial transcriptomics data into a common latent space (Fig. 3B). They have been extended to perform gene imputation on spatial transcriptomics data [36] and, likewise, could be adopted to perform spatial location construction for scRNA-seq data after the joint dimension reduction step.

## Cell-cell/gene-gene interactions

Cell signaling is constrained by physical location in the cellular microenvironment, as communicating cells are likely to be spatially adjacent. Integrating spatial information could potentially increase the accuracy of cell-cell communication inferences [28, 86], which is a typical application of spatial transcriptomics data analysis. To study cell-cell interactions, SVCA [31] utilizes Gaussian processes with additive covariance to model the variation of each gene’s expression. Specifically, SVCA [31] decomposes the variation in each gene into components of intrinsic, environmental, and cell-cell interaction effects. In particular, the cell-cell interaction effect is modeled by a covariance function integrating gene expression and spatial distances. SVCA [31] then calculates the proportion of variance attributable to the cell-cell interaction component through maximum likelihood with a gradient-based optimizer. If a gene’s variation is largely explained by the cell-cell interaction component, the cell may significantly interact with neighboring cells. GCNG [32] is a GCN-based model that encodes spatial information as a graph and combines it with the expression data as node features. Specifically, GCNG [32] first constructs an adjacency matrix from the spatial map by measuring cell-cell distances. Using the adjacency matrix and the ligand-receptor expression matrix as inputs, GCNG [32] utilizes two graph convolutional layers and a sigmoid function output layer for gene-gene interaction prediction. Notably, gene-gene interaction is often mediated by secreted cytokines, and interacting genes do not necessarily need to be adjacent to each other [84]. In this case, the two convolutional layers in GCNG [32] could detect these indirect interactions. Fischer et al. tackled the cell communication problem using node-centric expression modeling (NCEM), which is a graph neural networks based model [87]. MISTy [29] is a multiview model capable of learning interaction effects from both neighboring cells and distant cells. For a specific gene, MISTy [29] models its expression level as the output and other genes’ expression levels as the input. One of the views focuses on the local cellular niche and relates the expression from the immediate neighborhood of a cell to the observed expression within that cell. By analyzing how well different markers in this view contribute to predicting the target marker expression, we may identify potential interactions between the target marker and the predictor markers in a local spatial context. stLearn [30] and Squidpy [88] are pipelines that process and analyze spatial transcriptomics and tissue morphology data in an integrative manner and are capable of detecting cell-cell interactions. Specifically, stLearn [30] and Squidpy [88] utilize CellPhoneDB [89], a method proposed to study cell-cell interactions on scRNA-seq data using permutation tests, to identify ligand-receptor-mediated cell-cell interactions between identified cell clusters. Indeed, cellular spatial organizations are important for tissue functions and are mediated by ligand-receptor interactions [90, 91]. Theoretically, possible cell-cell communications or gene-gene interactions can be inferred using knowledge about spatially variable genes and ligand-receptor co-expression information. Such applications include novoSpaRc [56], SpaOTsc [55], and DEEPsc [39].

## Conclusions and perspectives

The fast development of spatial transcriptomics technology has spurred vast potentials for biological studies. However, the increasing data complexity due to additional spatial information has raised significant challenges for data analyses. As summarized in this review, different methods have been developed to tackle these challenges. Overall, spatial transcriptomics data analyses have benefited from integrating expression profiles with scRNA-seq data through joint dimension reduction [reviewed in [22]]. Commonly used methods for joint dimension reduction include NMF, PCA, SVD, CCA, and embeddings through convolutional networks. Among the computational methods that have been applied to spatial transcriptomics data, GCN is a promising tool and is gaining popularity [27, 32, 43], as it could leverage information from spatial neighborhoods to enhance data resolution. Likewise, GLISS [46], HMRF [47], and BayesSpace [48] also leverage information from neighboring cells to increase the sensitivity of localized expression pattern detection. On a separate note, semi-supervised learning utilizes both labeled and unlabeled data during model training and has proven to be effective in analyzing spatial transcriptomics data [27].

There exists an increasing number of resources for spatial transcriptomics research. SpatialDB [92] is a curated database for spatial transcriptomics datasets. It contains 24 datasets from 5 species generated by eight spatial transcriptomics techniques. In addition, the museum of spatial transcriptomics [12] provides a collection of study-level meta information of spatial transcriptomics datasets. Compared with spatial transcriptomics, scRNA-seq databases are more readily available. For example, TISCH [93] is a scRNA-seq database that has assembled transcriptome profiles of more than two million single cells. During the method development process, various spatial transcriptomics datasets have been generated or re-evaluated for benchmarking and performance evaluation. We have summarized different spatial transcriptomics datasets (Additional file 1: Table S1) and baseline methods (Additional file 1: Table S2) for method development in the papers that we have reviewed. In addition to the datasets, comprehensive pipelines to process the spatial transcriptomics data are available, including STUtility [94], Giotto [64], stLearn [30], dotdotdot [95], Squidpy [88], and GLISS [46]. These pipelines and toolboxes have covered a wide range of functions and algorithms to analyze and visualize spatial transcriptomics data. STUtility [94] takes 10X Genomics Visium data as the input and can perform data standardization, regional annotation, and visualization. Giotto [64] is a toolbox that implements algorithms for characterizing tissue composition, spatial expression pattern, and cellular interactions. stLearn [30] provides integrative approaches, including cell type annotation, cell pseudo-space-time reconstruction, and cell-cell interaction inference. Dotdotdot [95] is a computational workflow to preprocess spatial transcriptomics data and perform differential expression analysis. GLISS [46] could discover new spatial genes and recover cell locations in scRNA-seq data. These spatial transcriptomics datasets and analysis pipelines provide solid foundations for future method development for spatial transcriptomics data.

Challenges remain in the algorithm development for analyzing spatial transcriptomics data. As the field has achieved transcriptome-wide sequencing, spatial transcriptomics data quality is still limited by reduced coverage and low cellular resolution [96]. scRNA-seq has limitations of low capture efficiency and high dropouts, and these limitations are inherited by the spatial transcriptomics data [97]. In particular, in sequencing protocols where the size of the capture location is larger than a cell, multiple cells will be profiled as a mixture. In tumor microenvironment studies where immune cell infiltration is sparse and scattered, signals from the immune cells will be hardly captured since immune cells are dispersed. In addition, transcripts in spatial transcriptomics data do not necessarily follow a distribution similar to that of scRNA-seq data since these transcripts are from a mixture of multiple cells. Therefore, the assumptions made for analyzing scRNA-seq data need to be re-evaluated before applying to spatial transcriptomics research.

Multiple exciting directions remain to be explored in the field. As new computational algorithms are being developed rapidly, the field will benefit from more *systematic benchmark studies*, like what has been done for scRNA-seq data analyses [98–100]. To facilitate systematic benchmark studies, we have summarized the datasets that have been used for tool development and benchmarking (Additional file 1: Table S1) and the tools that have been used as baselines during method development (Additional file 1: Table S2) in the papers reviewed. Comprehensive benchmark studies could aid potential users in prioritizing the methods that best fit their data and hypotheses. Computational algorithms have been developed to *infer cell states and their developmental trajectories* in scRNA-seq data [reviewed in [18]]. Even though most of the knowledge we learned from trajectory inference for scRNA-seq data is applicable to spatial transcriptomics data, it is necessary to adapt the algorithms so that they can utilize spatial information effectively, as has been done in [101]. A method called scHOT [102] is a computational approach designed to identify changes in higher-order interactions among genes in cells along a continuous trajectory or across space. This method has also been demonstrated to be effective in spatial transcriptomics data. In addition, incorporation of spatial location information has the potential to increase the sensitivity of *cell-cell and gene-gene interaction studies*, as interacting cells are more likely to be spatially adjacent. Indeed, with the rapid expansion of ligand-receptor interaction and cytokine secretion related knowledge [103], *the integration of multiple data modalities* might open new opportunities to study cell-cell and gene-gene interactions, especially the multiway interactions that involve multiple parties. Furthermore, with the fast development of sequencing technology, high-throughput platforms for spatial multi-omics are becoming available, for example, SM-Omics could capture both spatially resolved transcriptomes and proteomes [104], whereas SHARE-seq measures high-throughput ATAC and RNA expression simultaneously [105]. By employing these platforms, more levels of molecular information will be collected from the same tissue section. These data will provide a more holistic view of the biological mechanisms and interactions but, at the same time, requires more sophisticated models with well-justified underlying assumptions for data analysis [106]. Additionally, it is important to transfer the multimodal data to spatial space for visualization at single-cell resolution. For this purpose, further method development is needed to incorporate the spatial information into the multi-omics data. The *construction of common coordinate frameworks (CCFs)* is a computational approach to integrate data from various sources into a consistent reference and to construct maps of molecular and cellular organization at histological and anatomical scales. The concept of CCFs has been discussed in [107], and the method development has been tackled in [108]. CCFs have also been generated with 3D reference, which can be used to analyze, visualize, and integrate multimodal and multiscale datasets in 3D. In terms of *3D modeling*, MERFISH has been extended to DNA imaging, which enables simultaneous imaging of the 3D organization of a tissue [109]. Computational approaches need to be developed to increase the efficiency of 3D data modeling and analysis. In addition, the analysis of spatial transcriptomics data from multiple tissue sections and time points has a potential to facilitate biological discovery, as has been done in [110]. Furthermore, neighboring cells in a tumor are likely to share similar copy number variations. Therefore, *copy number inference* on spatial transcriptomics data needs to be tackled, such as in [63, 111].

Spatial transcriptomics grants us a spatial perspective in addition to the expression data and hence allows for new angles to explore different areas of biological research. In this review, we surveyed the current advances in computational methods for integrating and analyzing spatial transcriptomics data, with a focus on the topics of localized gene expression pattern identification, spatial clustering, spatial decomposition, gene imputation, spatial location reconstruction, and cell-cell/gene-gene interaction inference. To aid future method development, we thoroughly summarized the datasets (Additional file 1: Table S1), baseline methods (Additional file 1: Table S2), and pipelines that are available for data preprocessing and benchmark studies. By highlighting the challenges and opportunities in this rapidly growing field, we anticipate motivating further studies to harness spatial transcriptomics data.

## Supplementary Information

**Additional file 1.** Supplementary tables (Table S1; Table S2). Summary of datasets and baseline methods for benchmark studies reviewed in the paper.

**Additional file 2.** Review history.

## Data Availability

Spatial transcriptomics benchmark datasets used by the computational methods reviewed in this paper are summarized in Table S1.
