# Supplementary material for: Statistical and machine learning methods for spatially resolved transcriptomics data analysis
Source: Genome Biol. 2022 Mar 25;23:83. doi: 10.1186/s13059-022-02653-7 (PMC8951701; doi:10.1186/s13059-022-02653-7)
Supplement: Supplementary file 1 — Additional file 1. Supplementary tables (Table S1; Table S2). Summary of datasets and baseline methods for benchmark studies reviewed in the paper. [file 13059_2022_2653_MOESM1_ESM.docx]

**Table S1. Spatial transcriptomics benchmark datasets used by the computational methods reviewed in this paper.** For each dataset, we curate the data information and referenced the original articles. We systematically profile the organism or disease from which the samples were derived, and the sequencing platforms used to generate the data. Furthermore, we curate the sources for data download.

| **NO.** | **Author** | **Organism/Disease** | **Sequencing Platform** | **Data Download** |
| --- | --- | --- | --- | --- |
| 1 | Stahl et al.[4] | 1. Mouse olfactory bulb 2. Human breast cancer | Spatial transcriptomics | 1. Sequencing data: https://www.ncbi.nlm.nih.gov/bioproject/?term=PRJNA316587 2. Gene counts: www.spatialtranscriptomicsresearch.org |
| 2 | Asp et al.[15] | Human heart | Spatial transcriptomics | 1. Sequencing data: https://ega-archive.org/studies/EGAS00001003996 2. Gene counts: https://www.spatialresearch.org/resources-published-datasets/doi-10-1016-j-cell-2019-11-025/ |
| 3 | Thrane et al.[112] | Cutaneous malignant melanoma | Spatial transcriptomics | https://www.spatialresearch.org/resources-published-datasets/doi-10-1158-0008-5472-can-18-0747/ |
| 4 | Maynard et al.[113] | Human dorsolateral prefrontal cortex | 10X Genomics Visium | http://research.libd.org/globus/ |
| 5 | 10X Genomic | Mouse posterior cerebrum, cerebellum and brainstem | 10X Genomics Visium | https://support.10xgenomics.com/spatial-gene-expression/datasets/1.0.0/V1_Mouse_Brain_Sagittal_Posterior |
| 6 | 10X Genomic | Mouse brain | 10X Genomics Visium | https://support.10xgenomics.com/spatial-gene-expression/datasets/1.1.0/V1_Adult_Mouse_Brain |
| 7 | 10X Genomic | Mouse brain | 10X Genomics Visium | https://support.10xgenomics.com/spatial-gene-expression/datasets/1.1.0/V1_Mouse_Brain_Sagittal_Anterior |
| 8 | 10X Genomic | Mouse kidney | 10X Genomics Visium | https://support.10xgenomics.com/spatial-gene-expression/datasets/1.1.0/V1_Mouse_Kidney |
| 9 | 10X Genomic | Human breast cancer | 10X Genomics Visium | section 1: https://support.10xgenomics.com/spatial-gene-expression/datasets/1.1.0/V1_Breast_Cancer_Block_A_Section_1 section 2: https://support.10xgenomics.com/spatial-gene-expression/datasets/1.1.0/V1_Breast_Cancer_Block_A_Section_2 |
| 10 | 10X Genomic | Human heart | 10X Genomics Visium | https://support.10xgenomics.com/spatial-gene-expression/datasets/1.1.0/V1_Human_Heart |
| 11 | 10X Genomic | Human lymph node | 10X Genomics Visium | https://support.10xgenomics.com/spatial-gene-expression/datasets/1.0.0/V1_Human_Lymph_Node |
| 12 | 10X Genomic | Human ovarian cancer | 10X Genomics Visium | https://support.10xgenomics.com/spatial-gene-expression/datasets/1.2.0/Targeted_Compare_Visium_Human_OvarianCancer_Immunology |
| 13 | Moncada et al.[114] | Human PDAC | Spatial transcriptomics | https://www.ncbi.nlm.nih.gov/geo/query/acc.cgi?acc=GSE111672 |
| 14 | Vickovic et al.[8] | Breast cancer and mouse brain | HDST | https://portals.broadinstitute.org/single_cell/study/SCP420 |
| 15 | Shah et al.[5] | Mouse hippocampus | seqFISH | Included in the paper |
| 16 | Eng et al.[7] | Cortex, subventricular zone and olfactory bulb of mouse brain | seqFISH | https://github.com/CaiGroup/seqFISH-PLUS |
| 17 | Zhu et al.[33] | Mouse visual cortex | seqFISH | http://spatial.rc.fas.harvard.edu/ |
| 18 | Moffitt et al.[115] | Mouse preoptic hypothalamus and surrounding areas of the brain | MERFISH | https://datadryad.org/stash/dataset/doi:10.5061/dryad.8t8s248 |
| 19 | Moffitt et al.[116] | Human osteosarcoma | MERFISH | Included in the paper |
| 20 | Xia et al.[117] | Human osteosarcoma | MERFISH | Included in the paper |
| 21 | Wang et al.^[56]^ | Mouse cortex | STARmap | Included in the paper |
| 22 | Codeluppi et al.[118] | Mouse somatosensory cortex | osmFISH | Available from the corresponding authors upon reasonable request |
| 23 | Stickels et al.[119] | Mouse hippocampus and neocortex | Slide-seqV2 | https://singlecell.broadinstitute.org/single_cell/study/SCP815/sensitive-spatial-genome-wide-expression-profiling-at-cellular-resolution#study-summary |
| 24 | Cable et al.[44] | Mouse cerebellum and somatosensory cortex | Slide-seqV2 | https://singlecell.broadinstitute.org/single_cell/study/SCP948 |
| 25 | Rodriques et al.[6] | Mouse brain | Slide-seq | https://singlecell.broadinstitute.org/single_cell/study/SCP354/slide-seq-study |
| 26 | Boisset et al.[83] | Mouse bone marrow | smFISH | Included in the paper |
| 27 | Halpern et al.[74] | Mouse liver | smFISH | Included in the paper |
| 28 | Satija et al.[67] | Zebrafish | ISH | Included in the paper |
| 29 | Karaiskos et al.[73] | Drosophila embryo | ISH | Included in the paper |
| 30 | Bageritz et al.[81] | Drosophila | ISH | Included in the paper |
| 31 | Joost et al.[75] | Murine telogen epidermis | FISH | Included in the paper |
| 32 | Berkeley Drosophila Transcription Network Project | Drosophila | FISH | http://bdtnp.lbl.gov:8080/Fly-Net/ |
| 33 | Achim et al.[72] | Brain of a marine annelid, P. dumerilii | WMISH | Included in the paper |
| 34 | Schapiro et al.[120] | Breast cancer | IMC | http://www.bodenmillerlab.org/research-2/histoCAT/ |
| 35 | Keren et al.[121] | Breast cancer | MIBI | https://mibi-share.ionpath.com |
| 36 | Goltsev et al.[122] | Mouse splenic | CODEX | https://data.mendeley.com/datasets/zjnpwh8m5b/1 |
| 37 | Long et al[123] | Caenorhabditis elegans | 3D digital | Included in the paper |
| 38 | Peng et al.[76] | Mid-gastrula mouse embryo | LCM and WISH | Included in the paper |
| 39 | Moor et al.[14] | Mouse intestinal villus axis | LCM-RNA-seq | https://www.ncbi.nlm.nih.gov/geo/query/acc.cgi?acc=GSE109413 |
| 40 | MOp data | Mouse primary motor cortex | MERFISH | https://doi.brainimagelibrary.org/doi/10.35077/g.21 |
| 41 | Berglund et al. [124] | Prostate cancer data | Spatial transcriptomics | 1. Gene counts: http://www.spatialtranscriptomicsresearch.org/ 2. Sequencing data: https://ega-archive.org/studies/EGAS00001003001 |
| 42 | Liu et al. [125] | Mouse embryo | DBiT-seq | https://www.ncbi.nlm.nih.gov/geo/query/acc.cgi?acc=GSE137986 |
| 43 | Mirzazadeh et al | Human small intestine | 10X Genomics Visium | https://data.mendeley.com/datasets/v8s9nz948s/1 |
| 44 | Abalo et al | Squamous cell carcinoma | 10X Genomics Visium | https://data.mendeley.com/datasets/2bh5fchcv6/1 |
| 45 | Ji et al. [126] | Human squamous cell carcinoma | 10X Genomics Visium | https://www.ncbi.nlm.nih.gov/geo/query/acc.cgi?acc=GSE144240 |
| 46 | Andersson et al. [127] | Human breast cancer | 10X Genomics Visium | https://zenodo.org/record/4751624#.Yflj7MjHjTI |

Abbreviations: HDST: high-definition spatial transcriptomics; smFISH: single-molecule fluorescence in-situ hybridization; LCM: saser capture microdissection; WISH: whole-mount in situ hybridization; CODEX: co-detection by indexing; MIBI: multiplexed ion beam imaging; IMC: imaging mass cytometry; WMISH: whole-mount in situ hybridization; ISH: in situ hybridization; FISH: fluorescence in situ hybridization; MERFISH: multiplexed error-robust fluorescence in situ hybridization; STARmap: spatially-resolved transcript amplicon readout mapping; seqFISH: sequential fluorescence in situ hybridization; PDAC: pancreatic ductal adenocarcinoma; DBiT-seq: deterministic barcoding in tissue for spatial omics sequencing

**Table S2. Computational methods used as baseline methods for benchmark studies during new method development.** Under each topic, we summarize the methods that each new method has used to establish its baselines for comparison. In addition, we summarize the datasets that have been used for method evaluation.

| **Method** | **Benchmarks** | **Spatial Transcriptomics Topics** | **Datasets in Table S1** |
| --- | --- | --- | --- |
| SpatialDWLS[43] | MuSiC[128], RCTD[44], SPOTlight[47], *stereoscope*[45] | Cell deconvolution | 2, 6, 16 |
| SPOTlight[47] | MuSiC[128], CIBERSORTx[129], DeconRNAseq[130], SCDC[131], RCTD[44], NMFreg[6] | Cell deconvolution | 13 |
| RCTD[44] | None | Cell deconvolution | 23, 24 |
| *stereoscope*[45] | deconvSeq[132], DWLS[42] | Cell deconvolution | 2, 25 |
| DSTG[46] | SPOTlight[47] | Cell deconvolution | 13, 23 |
| ProximID[83] | None | Cell-cell/gene-gene interactions | 26 |
| MISTy[86] | None | Cell-cell/gene-gene interactions | 9, 34 |
| Giotto[95] | None | Cell-cell/gene-gene interactions | 6, 8, 16, 18, 22, 21, 25, 35, 36 |
| stLearn[35] | None | 1. Spatial clustering  2. Cell-cell/gene-gene interactions | 14 |
| SVCA[84] | HistoCAT[120], Battich et al.[133], Goltsev et al.[122] | Cell-cell/gene-gene interactions | 15, 19, 34 |
| GCNG[85] | None | Cell-cell/gene-gene interactions | 16, 20 |
| Seurat[50] | Seurat[134], mnnCorrect[135], scanorama[136] | 1. Gene imputation 2. Spatial location reconstruction for scRNA-seq data | 21 |
| LIGER[58] | Seurat[50] | 1. Gene imputation 2. Spatial location reconstruction for scRNA-seq data | 21 |
| SpaGE[59] | Seurat[50], LIGER[58], gimVI[54] | 1. Gene imputation 2. Spatial location reconstruction for scRNA-seq data | 16, 18, 21, 22 |
| stPlus.[60] | SpaGE[59], Seurat[50], LIGER[58], gimVI[54] | Gene imputation | 16, 18, 22 |
| gimVI[54] | Seurat[50], LIGER[58], scVI[55], CORAL[137] | Gene imputation | 21, 22 |
| Harmony[57] | BBKNN[138], scanorama[136], mnnCorrect[135], Seurat[50] | 1. Gene imputation 2. Spatial location reconstruction for scRNA-seq data | 18 |
| Trendsceek[24] | None | Profiling of localized gene expression pattern | 1, 15 |
| SpatialDE[25] | None | Profiling of localized gene expression pattern | 1, 15, 18 |
| SPARK[27] | Trendsceek[24], SpatialDE[25] | 1. Profiling of localized gene expression pattern 2. Spatial location reconstruction for scRNA-seq data | 1, 15, 18 |
| SpaGCN[61] | Trendsceek[24], SpatialDE[25], SPARK[27] | 1. Profiling of localized gene expression pattern 2. Spatial Clustering  3. Spatial location reconstruction for scRNA-seq data | 1, 4, 5, 13, 18 |
| SPARK-X[29] | SPARK[27], SPARK-G[27], SpatialDE[25] | 1. Profiling of localized gene expression pattern 2. Spatial location reconstruction for scRNA-seq data | 10, 14, 23, 25 |
| *sepal*[30] | SpatialDE[25], SPARK[27] | 1. Profiling of localized gene expression pattern 2. Spatial location reconstruction for scRNA-seq data | 1, 3, 6, 11, 25 |
| Zhu et al[33] | None | 1. Profiling of localized gene expression pattern  2. Spatial clustering | 17 |
| BayesSpace[37] | Louvain[139], HMRF in Giotto[95], stLearn[35], mclust[140], SC3[141] | Spatial clustering | 3, 4, 12 |
| GLISS[32] | SpatialDE[25], scGCO[142], Trendsceek[24], Halpern et al.[74], Palantir[143], PAGA[144] | 1. Profiling of localized gene expression pattern 2. Spatial location reconstruction for scRNA-seq data | 1, 27, 39 |
| Seurat[67] | None | Spatial location reconstruction for scRNA-seq data | 28 |
| DEEPsc[77] | Achim et al.[72], Seurat[67], DistMap[73], Peng et al [76] | Gene imputation | 7, 28, 29 |
| CSOmap[79] | None | Spatial location reconstruction for scRNA-seq data | None |
| DistMap[73] | None | Spatial location reconstruction for scRNA-seq data | 29 |
| Halpern et al.[74] | None | Spatial location reconstruction for scRNA-seq data | 27 |
| Joost et al.[75] | None | Spatial location reconstruction for scRNA-seq data | 31 |
| Peng et al.[76] | None | Spatial location reconstruction for scRNA-seq data | 38 |
| Achim et al.[72] | None | Spatial location reconstruction for scRNA-seq data | 33 |
| Bageritz et al.[81] | None | Spatial location reconstruction for scRNA-seq data | 30 |
| SpaOTsc[65] | DistMap[73], Achim et al.[72], Seurat[67] | 1. Gene imputation 2. Cell-cell/gene-gene interactions | 16, 21, 25, 28, 29 |
| novoSpaRc[64] | None | 1. Gene imputation 2. Cell-cell/gene-gene interactions | 25, 32 |
| Tangram[48] | None | 1. Spatial location reconstruction for scRNA-seq data  2. Gene imputation | 21, 40 |
| SC-MEB[38] | K-means, GMM, BayesSpace[37], HMRF in Giotto[95] | Spatial clustering | 4, 18 |
| STAGAT[39] | BayesSpace[37], stLearn[35] | Spatial clustering | 4, 23, 25 |
| MULTILAYER[36] | SpatialDE [25], SPARK[27] | Spatial clustering | 1, 2, 13, 25, 41, 42 |
| Bergenstråhle et al.  [87] | No | Gene imputation | 1, 9, 43, 44 |
| HisToGene[69] | ST-Net[71] | Gene imputation | 45, 46 |
| STARCH[111] | InferCNV[145] | Gene imputation | 1 |

**REFERENCES AND NOTES**

1. Lubeck E, Cai L: **Single-cell systems biology by super-resolution imaging and combinatorial labeling.** *Nature methods* 2012, **9:**743-748.

2. Lubeck E, Coskun AF, Zhiyentayev T, Ahmad M, Cai L: **Single-cell in situ RNA profiling by sequential hybridization.** *Nature methods* 2014, **11:**360-361.

3. Chen KH, Boettiger AN, Moffitt JR, Wang S, Zhuang X: **Spatially resolved, highly multiplexed RNA profiling in single cells.** *Science* 2015, **348**.

4. Stahl PL, Salmen F, Vickovic S, Lundmark A, Navarro JF, Magnusson J, Giacomello S, Asp M, Westholm JO, Huss M, et al: **Visualization and analysis of gene expression in tissue sections by spatial transcriptomics.** *Science* 2016, **353:**78-82.

5. Shah S, Lubeck E, Zhou W, Cai L: **In situ transcription profiling of single cells reveals spatial organization of cells in the mouse hippocampus.** *Neuron* 2016, **92:**342-357.

6. Rodriques SG, Stickels RR, Goeva A, Martin CA, Murray E, Vanderburg CR, Welch J, Chen LM, Chen F, Macosko EZ: **Slide-seq: A scalable technology for measuring genome-wide expression at high spatial resolution.** *Science* 2019, **363:**1463-1467.

7. Eng C-HL, Lawson M, Zhu Q, Dries R, Koulena N, Takei Y, Yun J, Cronin C, Karp C, Yuan G-C: **Transcriptome-scale super-resolved imaging in tissues by RNA seqFISH+.** *Nature* 2019, **568:**235-239.

8. Vickovic S, Eraslan G, Salmén F, Klughammer J, Stenbeck L, Schapiro D, Äijö T, Bonneau R, Bergenstråhle L, Navarro JF: **High-definition spatial transcriptomics for in situ tissue profiling.** *Nature methods* 2019, **16:**987-990.

9. Bassiouni R, Gibbs LD, Craig DW, Carpten JD, McEachron TA: **Applicability of spatial transcriptional profiling to cancer research.** *Molecular Cell* 2021.

10. Zhuang X: **Spatially resolved single-cell genomics and transcriptomics by imaging.** *Nature Methods* 2021, **18:**18-22.

11. Crosetto N, Bienko M, Van Oudenaarden A: **Spatially resolved transcriptomics and beyond.** *Nature Reviews Genetics* 2015, **16:**57-66.

12. Moses L, Pachter L: **Museum of Spatial Transcriptomics.** *bioRxiv* 2021**:**2021.2005.2011.443152.

13. Ståhl PL, Salmén F, Vickovic S, Lundmark A, Navarro JF, Magnusson J, Giacomello S, Asp M, Westholm JO, Huss M: **Visualization and analysis of gene expression in tissue sections by spatial transcriptomics.** *Science* 2016, **353:**78-82.

14. Moor AE, Harnik Y, Ben-Moshe S, Massasa EE, Rozenberg M, Eilam R, Halpern KB, Itzkovitz S: **Spatial reconstruction of single enterocytes uncovers broad zonation along the intestinal villus axis.** *Cell* 2018, **175:**1156-1167.

15. Asp M, Giacomello S, Larsson L, Wu C, Fürth D, Qian X, Wärdell E, Custodio J, Reimegård J, Salmén F: **A spatiotemporal organ-wide gene expression and cell atlas of the developing human heart.** *Cell* 2019, **179:**1647-1660.

16. Fawkner-Corbett D, Antanaviciute A, Parikh K, Jagielowicz M, Gerós AS, Gupta T, Ashley N, Khamis D, Fowler D, Morrissey E: **Spatiotemporal analysis of human intestinal development at single-cell resolution.** *Cell* 2021, **184:**810-826.

17. Maniatis S, Petrescu J, Phatnani H: **Spatially resolved transcriptomics and its applications in cancer.** *Curr Opin Genet Dev* 2021, **66:**70-77.

18. Larsson L, Frisén J, Lundeberg J: **Spatially resolved transcriptomics adds a new dimension to genomics.** *Nature Methods* 2021, **18:**15-18.

19. Kharchenko PV: **The triumphs and limitations of computational methods for scRNA-seq.** *Nature Methods* 2021**:**1-10.

20. Lein E, Borm LE, Linnarsson S: **The promise of spatial transcriptomics for neuroscience in the era of molecular cell typing.** *Science* 2017, **358:**64-69.

21. Dries R, Chen J, Del Rossi N, Khan MM, Sistig A, Yuan G-C: **Advances in spatial transcriptomic data analysis.** *Genome Research* 2021, **31:**1706-1718.

22. Weiss K, Khoshgoftaar TM, Wang D: **A survey of transfer learning.** *Journal of Big Data* 2016, **3:**9.

23. Longo SK, Guo MG, Ji AL, Khavari PA: **Integrating single-cell and spatial transcriptomics to elucidate intercellular tissue dynamics.** *Nature Reviews Genetics* 2021**:**1-18.

24. Edsgärd D, Johnsson P, Sandberg R: **Identification of spatial expression trends in single-cell gene expression data.** *Nature methods* 2018, **15:**339-342.

25. Svensson V, Teichmann SA, Stegle O: **SpatialDE: identification of spatially variable genes.** *Nature methods* 2018, **15:**343-346.

26. Illian J, Penttinen A, Stoyan H, Stoyan D: *Statistical analysis and modelling of spatial point patterns.* John Wiley & Sons; 2008.

27. Sun S, Zhu J, Zhou X: **Statistical analysis of spatial expression patterns for spatially resolved transcriptomic studies.** *Nature methods* 2020, **17:**193-200.

28. Liu Y, Chen S, Li Z, Morrison AC, Boerwinkle E, Lin X: **ACAT: a fast and powerful p value combination method for rare-variant analysis in sequencing studies.** *The American Journal of Human Genetics* 2019, **104:**410-421.

29. Zhu J, Sun S, Zhou X: **SPARK-X: non-parametric modeling enables scalable and robust detection of spatial expression patterns for large spatial transcriptomic studies.** *Genome Biology* 2021, **22:**1-25.

30. Kats I, Vento-Tormo R, Stegle O: **SpatialDE2: Fast and localized variance component analysis of spatial transcriptomics.** *bioRxiv* 2021.

31. Andersson A, Lundeberg J: **sepal: identifying transcript profiles with spatial patterns by diffusion-based modeling.** *Bioinformatics* 2021.

32. Hu J, Li X, Coleman K, Schroeder A, Ma N, Irwin DJ, Lee EB, Shinohara RT, Li M: **SpaGCN: Integrating gene expression, spatial location and histology to identify spatial domains and spatially variable genes by graph convolutional network.** *Nature methods* 2021, **18:**1342-1351.

33. Zhu J, Sabatti C: **Integrative Spatial Single-cell Analysis with Graph-based Feature Learning.** *bioRxiv* 2020.

34. Zhu Q, Shah S, Dries R, Cai L, Yuan G-C: **Identification of spatially associated subpopulations by combining scRNAseq and sequential fluorescence in situ hybridization data.** *Nature biotechnology* 2018, **36:**1183-1190.

35. Moses L, Pachter L: **Museum of Spatial Transcriptomics.** 2021.

36. Pham D, Tan X, Xu J, Grice LF, Lam PY, Raghubar A, Vukovic J, Ruitenberg MJ, Nguyen Q: **stLearn: integrating spatial location, tissue morphology and gene expression to find cell types, cell-cell interactions and spatial trajectories within undissociated tissues.** *bioRxiv* 2020.

37. Moehlin J, Mollet B, Colombo BM, Mendoza-Parra MA: **Inferring biologically relevant molecular tissue substructures by agglomerative clustering of digitized spatial transcriptomes with multilayer.** *Cell Systems* 2021.

38. Zhao E, Stone MR, Ren X, Guenthoer J, Smythe KS, Pulliam T, Williams SR, Uytingco CR, Taylor SE, Nghiem P: **Spatial transcriptomics at subspot resolution with BayesSpace.** *Nature Biotechnology* 2021**:**1-10.

39. Yang Y, Shi X, Liu W, Zhou Q, Chan Lau M, Chun Tatt Lim J, Sun L, Ng CCY, Yeong J, Liu J: **SC-MEB: spatial clustering with hidden Markov random field using empirical Bayes.** *Briefings in bioinformatics* 2022, **23:**bbab466.

40. Dong K, Zhang S: **Deciphering spatial domains from spatially resolved transcriptomics with adaptive graph attention auto-encoder.** *bioRxiv* 2021.

41. Cobos FA, Alquicira-Hernandez J, Powell JE, Mestdagh P, De Preter K: **Benchmarking of cell type deconvolution pipelines for transcriptomics data.** *Nature communications* 2020, **11:**1-14.

42. Sturm G, Finotello F, Petitprez F, Zhang JD, Baumbach J, Fridman WH, List M, Aneichyk T: **Comprehensive evaluation of transcriptome-based cell-type quantification methods for immuno-oncology.** *Bioinformatics* 2019, **35:**i436-i445.

43. Tsoucas D, Dong R, Chen H, Zhu Q, Guo G, Yuan G-C: **Accurate estimation of cell-type composition from gene expression data.** *Nature communications* 2019, **10:**1-9.

44. Dong R, Yuan GC: **SpatialDWLS: accurate deconvolution of spatial transcriptomic data.** *Genome Biol* 2021, **22:**145.

45. Elosua-Bayes M, Nieto P, Mereu E, Gut I, Heyn H: **SPOTlight: seeded NMF regression to deconvolute spatial transcriptomics spots with single-cell transcriptomes.** *Nucleic Acids Res* 2021.

46. Cable DM, Murray E, Zou LS, Goeva A, Macosko EZ, Chen F, Irizarry RA: **Robust decomposition of cell type mixtures in spatial transcriptomics.** *Nature Biotechnology* 2021**:**1-10.

47. Andersson A, Bergenstrahle J, Asp M, Bergenstrahle L, Jurek A, Fernandez Navarro J, Lundeberg J: **Single-cell and spatial transcriptomics enables probabilistic inference of cell type topography.** *Commun Biol* 2020, **3:**565.

48. Song Q, Su J: **DSTG: deconvoluting spatial transcriptomics data through graph-based artificial intelligence.** *Briefings in Bioinformatics* 2021.

49. Biancalani T, Scalia G, Buffoni L, Avasthi R, Lu Z, Sanger A, Tokcan N, Vanderburg CR, Segerstolpe Å, Zhang M: **Deep learning and alignment of spatially resolved single-cell transcriptomes with Tangram.** *Nature methods* 2021, **18:**1352-1362.

50. Kleshchevnikov V, Shmatko A, Dann E, Aivazidis A, King HW, Li T, Lomakin A, Kedlian V, Jain MS, Park JS: **Comprehensive mapping of tissue cell architecture via integrated single cell and spatial transcriptomics.** *bioRxiv* 2020.

51. Stuart T, Butler A, Hoffman P, Hafemeister C, Papalexi E, Mauck Iii WM, Hao Y, Stoeckius M, Smibert P, Satija R: **Comprehensive integration of single-cell data.** *Cell* 2019, **177:**1888-1902.

52. Aliee H, Theis FJ: **AutoGeneS: Automatic gene selection using multi-objective optimization for RNA-seq deconvolution.** *Cell Systems* 2021.

53. Elosua-Bayes M, Nieto P, Mereu E, Gut I, Heyn H: **SPOTlight: seeded NMF regression to deconvolute spatial transcriptomics spots with single-cell transcriptomes.** *Nucleic acids research* 2021, **49:**e50-e50.

54. Moncada R, Barkley D, Wagner F, Chiodin M, Devlin JC, Baron M, Hajdu CH, Simeone DM, Yanai I: **Integrating microarray-based spatial transcriptomics and single-cell RNA-seq reveals tissue architecture in pancreatic ductal adenocarcinomas.** *Nature Biotechnology* 2020, **38:**333-342.

55. Lopez R, Nazaret A, Langevin M, Samaran J, Regier J, Jordan M, Yosef N: *A joint model of unpaired data from scRNA-seq and spatial transcriptomics for imputing missing gene expression measurements.* 2019.

56. Lopez R, Regier J, Cole MB, Jordan MI, Yosef N: **Deep generative modeling for single-cell transcriptomics.** *Nature methods* 2018, **15:**1053-1058.

57. Wang X, Allen WE, Wright MA, Sylwestrak EL, Samusik N, Vesuna S, Evans K, Liu C, Ramakrishnan C, Liu J: **Three-dimensional intact-tissue sequencing of single-cell transcriptional states.** *Science* 2018, **361**.

58. Korsunsky I, Millard N, Fan J, Slowikowski K, Zhang F, Wei K, Baglaenko Y, Brenner M, Loh P-r, Raychaudhuri S: **Fast, sensitive and accurate integration of single-cell data with Harmony.** *Nature methods* 2019, **16:**1289-1296.

59. Welch JD, Kozareva V, Ferreira A, Vanderburg C, Martin C, Macosko EZ: **Single-Cell Multi-omic Integration Compares and Contrasts Features of Brain Cell Identity.** *Cell* 2019, **177:**1873-1887 e1817.

60. Abdelaal T, Mourragui S, Mahfouz A, Reinders MJT: **SpaGE: spatial gene enhancement using scRNA-seq.** *Nucleic acids research* 2020, **48:**e107-e107.

61. Shengquan C, Boheng Z, Xiaoyang C, Xuegong Z, Rui J: **stPlus: a reference-based method for the accurate enhancement of spatial transcriptomics.** *Bioinformatics* 2021, **37:**i299-i307.

62. Hu J, Li X, Coleman K, Schroeder A, Irwin DJ, Lee EB, Shinohara RT, Li M: **Integrating gene expression, spatial location and histology to identify spatial domains and spatially variable genes by graph convolutional network.** *bioRxiv* 2020**:**2020.2011.2030.405118.

63. Zhao E, Stone MR, Ren X, Guenthoer J, Smythe KS, Pulliam T, Williams SR, Uytingco CR, Taylor SEB, Nghiem P: **Spatial transcriptomics at subspot resolution with BayesSpace.** *Nature Biotechnology* 2021**:**1-10.

64. Villani C: *Optimal transport: old and new.* Springer; 2009.

65. Nitzan M, Karaiskos N, Friedman N, Rajewsky N: **Gene expression cartography.** *Nature* 2019, **576:**132-137.

66. Cang Z, Nie Q: **Inferring spatial and signaling relationships between cells from single cell transcriptomic data.** *Nature communications* 2020, **11:**1-13.

67. Qian X, Harris KD, Hauling T, Nicoloutsopoulos D, Muñoz-Manchado AB, Skene N, Hjerling-Leffler J, Nilsson M: **Probabilistic cell typing enables fine mapping of closely related cell types in situ.** *Nature methods* 2020, **17:**101-106.

68. Satija R, Farrell JA, Gennert D, Schier AF, Regev A: **Spatial reconstruction of single-cell gene expression data.** *Nature biotechnology* 2015, **33:**495-502.

69. Bergenstråhle L, He B, Bergenstråhle J, Abalo X, Mirzazadeh R, Thrane K, Ji AL, Andersson A, Larsson L, Stakenborg N: **Super-resolved spatial transcriptomics by deep data fusion.** *Nature biotechnology* 2021**:**1-4.

70. Pang M, Su K, Li M: **Leveraging information in spatial transcriptomics to predict super-resolution gene expression from histology images in tumors.** *bioRxiv* 2021.

71. Schmauch B, Romagnoni A, Pronier E, Saillard C, Maillé P, Calderaro J, Kamoun A, Sefta M, Toldo S, Zaslavskiy M: **A deep learning model to predict RNA-Seq expression of tumours from whole slide images.** *Nature communications* 2020, **11:**1-15.

72. He B, Bergenstråhle L, Stenbeck L, Abid A, Andersson A, Borg Å, Maaskola J, Lundeberg J, Zou J: **Integrating spatial gene expression and breast tumour morphology via deep learning.** *Nature biomedical engineering* 2020, **4:**827-834.

73. Achim K, Pettit J-B, Saraiva LR, Gavriouchkina D, Larsson T, Arendt D, Marioni JC: **High-throughput spatial mapping of single-cell RNA-seq data to tissue of origin.** *Nature biotechnology* 2015, **33:**503-509.

74. Karaiskos N, Wahle P, Alles J, Boltengagen A, Ayoub S, Kipar C, Kocks C, Rajewsky N, Zinzen RP: **The Drosophila embryo at single-cell transcriptome resolution.** *Science* 2017, **358:**194-199.

75. Halpern KB, Shenhav R, Matcovitch-Natan O, Toth B, Lemze D, Golan M, Massasa EE, Baydatch S, Landen S, Moor AE: **Single-cell spatial reconstruction reveals global division of labour in the mammalian liver.** *Nature* 2017, **542:**352-356.

76. Joost S, Zeisel A, Jacob T, Sun X, La Manno G, Lönnerberg P, Linnarsson S, Kasper M: **Single-cell transcriptomics reveals that differentiation and spatial signatures shape epidermal and hair follicle heterogeneity.** *Cell systems* 2016, **3:**221-237.

77. Peng G, Suo S, Chen J, Chen W, Liu C, Yu F, Wang R, Chen S, Sun N, Cui G: **Spatial transcriptome for the molecular annotation of lineage fates and cell identity in mid-gastrula mouse embryo.** *Developmental cell* 2016, **36:**681-697.

78. Maseda F, Cang Z, Nie Q: **DEEPsc: A Deep Learning-Based Map Connecting Single-Cell Transcriptomics and Spatial Imaging Data.** *Front Genet* 2021, **12:**636743.

79. Chizat L, Peyré G, Schmitzer B, Vialard F-X: **Scaling algorithms for unbalanced optimal transport problems.** *Mathematics of Computation* 2018, **87:**2563-2609.

80. Ren X, Zhong G, Zhang Q, Zhang L, Sun Y, Zhang Z: **Reconstruction of cell spatial organization from single-cell RNA sequencing data based on ligand-receptor mediated self-assembly.** *Cell research* 2020, **30:**763-778.

81. Ramilowski JA, Goldberg T, Harshbarger J, Kloppmann E, Lizio M, Satagopam VP, Itoh M, Kawaji H, Carninci P, Rost B: **A draft network of ligand–receptor-mediated multicellular signalling in human.** *Nature communications* 2015, **6:**1-12.

82. Bageritz J, Willnow P, Valentini E, Leible S, Boutros M, Teleman AA: **Gene expression atlas of a developing tissue by single cell expression correlation analysis.** *Nature methods* 2019, **16:**750-756.

83. Almet AA, Cang Z, Jin S, Nie Q: **The landscape of cell-cell communication through single-cell transcriptomics.** *Current Opinion in Systems Biology* 2021.

84. Boisset J-C, Vivié J, Grün D, Muraro MJ, Lyubimova A, Van Oudenaarden A: **Mapping the physical network of cellular interactions.** *Nature methods* 2018, **15:**547-553.

85. Arnol D, Schapiro D, Bodenmiller B, Saez-Rodriguez J, Stegle O: **Modeling cell-cell interactions from spatial molecular data with spatial variance component analysis.** *Cell reports* 2019, **29:**202-211. e206.

86. Yuan Y, Bar-Joseph Z: **GCNG: graph convolutional networks for inferring gene interaction from spatial transcriptomics data.** *Genome Biology* 2020, **21:**1-16.

87. Fischer DS, Schaar AC, Theis FJ: **Learning cell communication from spatial graphs of cells.** *bioRxiv* 2021.

88. Tanevski J, Gabor A, Flores ROR, Schapiro D, Saez-Rodriguez J: **Explainable multi-view framework for dissecting inter-cellular signaling from highly multiplexed spatial data.** *bioRxiv* 2020.

89. Pham DT, Tan X, Xu J, Grice LF, Lam PY, Raghubar A, Vukovic J, Ruitenberg MJ, Nguyen QH: **stLearn: integrating spatial location, tissue morphology and gene expression to find cell types, cell-cell interactions and spatial trajectories within undissociated tissues.** *bioRxiv* 2020.

90. Palla G, Spitzer H, Klein M, Fischer D, Schaar AC, Kuemmerle LB, Rybakov S, Ibarra IL, Holmberg O, Virshup I: **Squidpy: a scalable framework for spatial single cell analysis.** *bioRxiv* 2021.

91. Garcia-Alonso L, Handfield LF, Roberts K, Nikolakopoulou K, Fernando RC, Gardner L, Woodhams B, Arutyunyan A, Polanski K, Hoo R, et al: **Mapping the temporal and spatial dynamics of the human endometrium in vivo and in vitro.** *Nat Genet* 2021, **53:**1698-1711.

92. Beccari L, Moris N, Girgin M, Turner DA, Baillie-Johnson P, Cossy A-C, Lutolf MP, Duboule D, Arias AM: **Multi-axial self-organization properties of mouse embryonic stem cells into gastruloids.** *Nature* 2018, **562:**272-276.

93. Toda S, Blauch LR, Tang SKY, Morsut L, Lim WA: **Programming self-organizing multicellular structures with synthetic cell-cell signaling.** *Science* 2018, **361:**156-162.

94. Fan Z, Chen R, Chen X: **SpatialDB: a database for spatially resolved transcriptomes.** *Nucleic acids research* 2020, **48:**D233-D237.

95. Sun D, Wang J, Han Y, Dong X, Ge J, Zheng R, Shi X, Wang B, Li Z, Ren P: **TISCH: a comprehensive web resource enabling interactive single-cell transcriptome visualization of tumor microenvironment.** *Nucleic acids research* 2021, **49:**D1420-D1430.

96. Bergenstråhle J, Larsson L, Lundeberg J: **Seamless integration of image and molecular analysis for spatial transcriptomics workflows.** *BMC genomics* 2020, **21:**1-7.

97. Dries R, Zhu Q, Dong R, Eng C-HL, Li H, Liu K, Fu Y, Zhao T, Sarkar A, Bao F: **Giotto: a toolbox for integrative analysis and visualization of spatial expression data.** *Genome biology* 2021, **22:**1-31.

98. Maynard KR, Tippani M, Takahashi Y, Phan BN, Hyde TM, Jaffe AE, Martinowich K: **dotdotdot: an automated approach to quantify multiplex single molecule fluorescent in situ hybridization (smFISH) images in complex tissues.** *Nucleic acids research* 2020, **48:**e66-e66.

99. Waylen LN, Nim HT, Martelotto LG, Ramialison M: **From whole-mount to single-cell spatial assessment of gene expression in 3D.** *Communications biology* 2020, **3:**1-11.

100. Haque A, Engel J, Teichmann SA, Lönnberg T: **A practical guide to single-cell RNA-sequencing for biomedical research and clinical applications.** *Genome medicine* 2017, **9:**1-12.

101. Abdelaal T, Michielsen L, Cats D, Hoogduin D, Mei H, Reinders MJT, Mahfouz A: **A comparison of automatic cell identification methods for single-cell RNA sequencing data.** *Genome biology* 2019, **20:**1-19.

102. Tran HTN, Ang KS, Chevrier M, Zhang X, Lee NYS, Goh M, Chen J: **A benchmark of batch-effect correction methods for single-cell RNA sequencing data.** *Genome biology* 2020, **21:**1-32.

103. Xi NM, Li JJ: **Benchmarking computational doublet-detection methods for single-cell rna sequencing data.** *Cell systems* 2021, **12:**176-194.

104. Xia C, Fan J, Emanuel G, Hao J, Zhuang X: **Spatial transcriptome profiling by MERFISH reveals subcellular RNA compartmentalization and cell cycle-dependent gene expression.** *Proceedings of the National Academy of Sciences* 2019, **116:**19490-19499.

105. Ghazanfar S, Lin Y, Su X, Lin DM, Patrick E, Han Z-G, Marioni JC, Yang JYH: **Investigating higher-order interactions in single-cell data with scHOT.** *Nature methods* 2020, **17:**799-806.

106. Jiang P, Zhang Y, Ru B, Yang Y, Vu T, Paul R, Mirza A, Altan-Bonnet G, Liu L, Ruppin E: **Systematic investigation of cytokine signaling activity at the tissue and single-cell levels.** *Nature Methods* 2021, **18:**1181-1191.

107. Vickovic S, Loetstedt B, Klughammer J, Segerstolpe A, Rozenblatt-Rosen O, Regev A: **SM-Omics: An automated platform for high-throughput spatial multi-omics.** *bioRxiv* 2020.

108. Ma S, Zhang B, LaFave LM, Earl AS, Chiang Z, Hu Y, Ding J, Brack A, Kartha VK, Tay T: **Chromatin potential identified by shared single-cell profiling of RNA and chromatin.** *Cell* 2020, **183:**1103-1116.

109. Deng Y, Bartosovic M, Kukanja P, Zhang D, Liu Y, Su G, Enninful A, Bai Z, Castelo-Branco G, Fan R: **Spatial-CUT&Tag: Spatially resolved chromatin modification profiling at the cellular level.** *Science* 2022, **375:**681-686.

110. Rood JE, Stuart T, Ghazanfar S, Biancalani T, Fisher E, Butler A, Hupalowska A, Gaffney L, Mauck W, Eraslan G: **Toward a common coordinate framework for the human body.** *Cell* 2019, **179:**1455-1467.

111. Andersson A, Andrusivová Ž, Czarnewski P, Li X, Sundström E, Lundeberg J: **A landmark-based common coordinate framework for spatial transcriptomics data.** *bioRxiv* 2021.

112. Su J-H, Zheng P, Kinrot SS, Bintu B, Zhuang X: **Genome-scale imaging of the 3D organization and transcriptional activity of chromatin.** *Cell* 2020, **182:**1641-1659.

113. Äijö T, Maniatis S, Vickovic S, Kang K, Cuevas M, Braine C, Phatnani H, Lundeberg J, Bonneau R: **Splotch: Robust estimation of aligned spatial temporal gene expression data.** *bioRxiv* 2019**:**757096.

114. Elyanow R, Zeira R, Land M, Raphael BJ: **STARCH: Copy number and clone inference from spatial transcriptomics data.** *Physical Biology* 2021, **18:**035001.

115. Erickson A, Berglund E, He M, Marklund M, Mirzazadeh R, Schultz N, Bergenstråhle L, Kvastad L, Andersson A, Bergenstråhle J: **The spatial landscape of clonal somatic mutations in benign and malignant tissue.** *bioRxiv* 2021.

116. Thrane K, Eriksson H, Maaskola J, Hansson J, Lundeberg J: **Spatially Resolved Transcriptomics Enables Dissection of Genetic Heterogeneity in Stage III Cutaneous Malignant Melanoma.** *Cancer Res* 2018, **78:**5970-5979.

117. Maynard KR, Collado-Torres L, Weber LM, Uytingco C, Barry BK, Williams SR, Catallini JL, 2nd, Tran MN, Besich Z, Tippani M, et al: **Transcriptome-scale spatial gene expression in the human dorsolateral prefrontal cortex.** *Nat Neurosci* 2021, **24:**425-436.

118. Moncada R, Barkley D, Wagner F, Chiodin M, Devlin JC, Baron M, Hajdu CH, Simeone DM, Yanai I: **Integrating microarray-based spatial transcriptomics and single-cell RNA-seq reveals tissue architecture in pancreatic ductal adenocarcinomas.** *Nat Biotechnol* 2020, **38:**333-342.

119. Moffitt JR, Bambah-Mukku D, Eichhorn SW, Vaughn E, Shekhar K, Perez JD, Rubinstein ND, Hao J, Regev A, Dulac C, Zhuang X: **Molecular, spatial, and functional single-cell profiling of the hypothalamic preoptic region.** *Science* 2018, **362**.

120. Moffitt JR, Hao J, Wang G, Chen KH, Babcock HP, Zhuang X: **High-throughput single-cell gene-expression profiling with multiplexed error-robust fluorescence in situ hybridization.** *Proc Natl Acad Sci U S A* 2016, **113:**11046-11051.

121. Xia C, Fan J, Emanuel G, Hao J, Zhuang X: **Spatial transcriptome profiling by MERFISH reveals subcellular RNA compartmentalization and cell cycle-dependent gene expression.** *Proc Natl Acad Sci U S A* 2019, **116:**19490-19499.

122. Codeluppi S, Borm LE, Zeisel A, La Manno G, van Lunteren JA, Svensson CI, Linnarsson S: **Spatial organization of the somatosensory cortex revealed by osmFISH.** *Nat Methods* 2018, **15:**932-935.

123. Stickels RR, Murray E, Kumar P, Li J, Marshall JL, Di Bella DJ, Arlotta P, Macosko EZ, Chen F: **Highly sensitive spatial transcriptomics at near-cellular resolution with Slide-seqV2.** *Nat Biotechnol* 2021, **39:**313-319.

124. Schapiro D, Jackson HW, Raghuraman S, Fischer JR, Zanotelli VRT, Schulz D, Giesen C, Catena R, Varga Z, Bodenmiller B: **histoCAT: analysis of cell phenotypes and interactions in multiplex image cytometry data.** *Nat Methods* 2017, **14:**873-876.

125. Keren L, Bosse M, Marquez D, Angoshtari R, Jain S, Varma S, Yang SR, Kurian A, Van Valen D, West R, et al: **A Structured Tumor-Immune Microenvironment in Triple Negative Breast Cancer Revealed by Multiplexed Ion Beam Imaging.** *Cell* 2018, **174:**1373-1387 e1319.

126. Goltsev Y, Samusik N, Kennedy-Darling J, Bhate S, Hale M, Vazquez G, Black S, Nolan GP: **Deep Profiling of Mouse Splenic Architecture with CODEX Multiplexed Imaging.** *Cell* 2018, **174:**968-981 e915.

127. Long F, Peng H, Liu X, Kim SK, Myers E: **A 3D digital atlas of C. elegans and its application to single-cell analyses.** *Nat Methods* 2009, **6:**667-672.

128. Berglund E, Maaskola J, Schultz N, Friedrich S, Marklund M, Bergenstråhle J, Tarish F, Tanoglidi A, Vickovic S, Larsson L: **Spatial maps of prostate cancer transcriptomes reveal an unexplored landscape of heterogeneity.** *Nature communications* 2018, **9:**1-13.

129. Liu Y, Yang M, Deng Y, Su G, Enninful A, Guo CC, Tebaldi T, Zhang D, Kim D, Bai Z: **High-spatial-resolution multi-omics sequencing via deterministic barcoding in tissue.** *Cell* 2020, **183:**1665-1681.

130. Ji AL, Rubin AJ, Thrane K, Jiang S, Reynolds DL, Meyers RM, Guo MG, George BM, Mollbrink A, Bergenstråhle J: **Multimodal analysis of composition and spatial architecture in human squamous cell carcinoma.** *Cell* 2020, **182:**497-514.

131. Andersson A, Larsson L, Stenbeck L, Salmén F, Ehinger A, Wu SZ, Al-Eryani G, Roden D, Swarbrick A, Borg Å: **Spatial deconvolution of HER2-positive breast cancer delineates tumor-associated cell type interactions.** *Nature communications* 2021, **12:**1-14.

132. Wang X, Park J, Susztak K, Zhang NR, Li M: **Bulk tissue cell type deconvolution with multi-subject single-cell expression reference.** *Nat Commun* 2019, **10:**380.

133. Newman AM, Steen CB, Liu CL, Gentles AJ, Chaudhuri AA, Scherer F, Khodadoust MS, Esfahani MS, Luca BA, Steiner D, et al: **Determining cell type abundance and expression from bulk tissues with digital cytometry.** *Nat Biotechnol* 2019, **37:**773-782.

134. Gong T, Szustakowski JD: **DeconRNASeq: a statistical framework for deconvolution of heterogeneous tissue samples based on mRNA-Seq data.** *Bioinformatics* 2013, **29:**1083-1085.

135. Dong M, Thennavan A, Urrutia E, Li Y, Perou CM, Zou F, Jiang Y: **SCDC: bulk gene expression deconvolution by multiple single-cell RNA sequencing references.** *Brief Bioinform* 2021, **22:**416-427.

136. Du R, Carey V, Weiss ST: **deconvSeq: deconvolution of cell mixture distribution in sequencing data.** *Bioinformatics* 2019, **35:**5095-5102.

137. Battich N, Stoeger T, Pelkmans L: **Control of Transcript Variability in Single Mammalian Cells.** *Cell* 2015, **163:**1596-1610.

138. Butler A, Hoffman P, Smibert P, Papalexi E, Satija R: **Integrating single-cell transcriptomic data across different conditions, technologies, and species.** *Nat Biotechnol* 2018, **36:**411-420.

139. Haghverdi L, Lun ATL, Morgan MD, Marioni JC: **Batch effects in single-cell RNA-sequencing data are corrected by matching mutual nearest neighbors.** *Nat Biotechnol* 2018, **36:**421-427.

140. Hie B, Bryson B, Berger B: **Efficient integration of heterogeneous single-cell transcriptomes using Scanorama.** *Nat Biotechnol* 2019, **37:**685-691.

141. Sun B, Feng J, Saenko K: **Return of frustratingly easy domain adaptation.** In *Proceedings of the Thirtieth AAAI Conference on Artificial Intelligence*. pp. 2058–2065. Phoenix, Arizona: AAAI Press; 2016:2058–2065.

142. Polanski K, Young MD, Miao Z, Meyer KB, Teichmann SA, Park JE: **BBKNN: fast batch alignment of single cell transcriptomes.** *Bioinformatics* 2020, **36:**964-965.

143. Blondel VD, Guillaume J-L, Lambiotte R, Lefebvre E: **Fast unfolding of communities in large networks.** *Journal of Statistical Mechanics: Theory and Experiment* 2008, **2008:**P10008.

144. Fraley C, Raftery A, Murphy T, Scrucca L: **MCLUST Version 4 for R: Normal Mixture Modeling for Model-Based Clustering, Classification, and Density Estimation.** *Technical Report No 597* 2012.

145. Kiselev VY, Kirschner K, Schaub MT, Andrews T, Yiu A, Chandra T, Natarajan KN, Reik W, Barahona M, Green AR, Hemberg M: **SC3: consensus clustering of single-cell RNA-seq data.** *Nat Methods* 2017, **14:**483-486.

146. Zhang K, Feng W, Wang P: **Identification of spatially variable genes with graph cuts.** *bioRxiv* 2018**:**491472.

147. Setty M, Kiseliovas V, Levine J, Gayoso A, Mazutis L, Pe'er D: **Characterization of cell fate probabilities in single-cell data with Palantir.** *Nat Biotechnol* 2019, **37:**451-460.

148. Wolf FA, Hamey FK, Plass M, Solana J, Dahlin JS, Gottgens B, Rajewsky N, Simon L, Theis FJ: **PAGA: graph abstraction reconciles clustering with trajectory inference through a topology preserving map of single cells.** *Genome Biol* 2019, **20:**59.

149. Tirosh I, Izar B, Prakadan SM, Wadsworth MH, Treacy D, Trombetta JJ, Rotem A, Rodman C, Lian C, Murphy G: **Dissecting the multicellular ecosystem of metastatic melanoma by single-cell RNA-seq.** *Science* 2016, **352:**189-196.
