# Supplementary material for: Statistical and machine learning methods for spatially resolved transcriptomics data analysis
Source: Genome Biol. 2022 Mar 25;23:83. doi: 10.1186/s13059-022-02653-7 (PMC8951701; doi:10.1186/s13059-022-02653-7)
Supplement: Supplementary file 2 — Additional file 2. Review history. [file 13059_2022_2653_MOESM2_ESM.docx]

Review history

**First round of review**

**Reviewer 1:**

In the manuscript entitled "Statistical and Machine Learning Methods for Spatially Resolved Transcriptomics Data Analysis", Zeng et al. mainly summarized the current statistical and machine learning methods for spatial transcriptomics data analysis with several different research areas, including gene expression spatial pattern identification, spatial transcriptomics data quality improvement (such as cell deconvolution and gene imputation), space reconstruction for scRNA-seq data, and cell-cell/gene-gene interactions. Also, they provided a well-organized spatial transcriptomics data set and baseline methods list, which could be useful for further developing methods for spatial transcriptomics data analysis.
Considering the rapid advancement and great power of spatial transcriptomics technology, summarizing this review about methods for spatial transcriptomics data analysis is meaningful and can help lots of researchers. However, this review mainly provided the brief introduction of different computational methods, lacking the systematic organization and summary of them. Therefore, I don't think the current version of this review meet the qualification for publication.
My detailed comments are listed below and I hoped they would be helpful for the authors to further improve the manuscript.

1. The development background as well as interconnection of these types of methods covered in the review were not clearly described. For instance, though "cellular deconvolution" and "gene imputation" are both tools developed to improve data quality, they were specifical for two different types of spatial transcriptomics data. Also, space reconstruction is a unique analysis to predict spatial information for scRNA-seq data, which is quite different from other analysis of spatial transcriptomics data. For a logical review the authors would need to summarize all the methods according to the standard workflow of spatial transcriptomics data analysis.
2. For all the methods mentioned in this review, I think it would be clearer to list the principles, application scenarios, advantages and disadvantages of them into a table. Only simply describing how these methods work doesn't truly give useful guide to researchers.
3. In the subsection "profiling of localized gene expression pattern", I only found the Trendsceek-liked and GCN-based methods in Fig.2. Why other machine learning methods such as sepal mentioned in the article were not showed in Fig.2? Additionally, SpaGCN and BayesSpace provided a spatial-clustering step, which is also an important research area in spatial transcriptomics data analysis. Therefore, it may be better to review other spatial clustering methods in a separate subsection.
4. In the subsection "cellular deconvolution" and "gene imputation", methods with the same principles were supposed to be summarized together. For example, both spatialDWLS and SPOTlight deconvolute the spots of spatial transcriptomics data based on statistical regression, and describing them separately would cause confusion.
5. In the subsection "conclusions and perspectives", due to most of methods have been reviewed in former subsection, summarizing them in detail again seemed not necessary. Moreover, the perspective of authors was not summarized clearly.

**Reviewer 2:**

General summary

The authors set out to give a comprehensive account of the computational methods that exist for analysis of spatial transcriptomics data, as well as outlining some of the challenges that still remains to be addressed in the field. They list five broad main categories into which current techniques fall, being:

• Profiling of localized gene expression pattern

• Cellular deconvolution

• Gene Imputation

• Spatial location reconstruction for scRNA-seq data

• Cell-cell/gene-gene interactions

For each of these categories, they describe the objective of the associated methods and give examples of different strategies to achieve this. The work also contains a discussion of general pipelines for analysis of spatial transcriptomics data as well as efforts to gather data sets.

General comments

Personally, I found the manuscript well structured and easy to read. The authors have written it in a short a concise format that makes it easy for the reader to get a "bird’s eye view" of the landscape of different computational methods. For the five topics they choose as overarching categories, they provide fairly extensive record of existing methods. However, there are some areas that if given additional attention, would elevate the quality of this manuscript even further. In general, I do feel as if some "historical" context is lacking with certain earlier methods not being mentioned or credited. While I fully understand that current methods are more relevant to the reader, I also believe that early contributors who "pioneered" the ideas should be mentioned; especially since these early methods often rely on simpler approaches that many readers may have contemplated themselves. The authors also mention that many of the techniques developed for analysis of single cell data could be and are applied to spatial data, but don’t really give examples of such analysis. These methods are not inherently spatial, but as they are commonly employed by researchers I also think mentioning them would be worthwhile. I would also try to discuss some additional flavors of methods in order to give a more comprehensive overview, potentially expanding the five categories (more on this later).

Additionally, I have some comments regarding the statements and formulations used in the manuscript that I’ve taken the liberty to share with the authors, although I would not consider these as a requirement for my satisfaction, but rather just tips or input from someone with a different perspective. Finally, I would have preferred to see a slightly more explicit account of the future challenges, here I have some suggestions – which I by no means want to enforce upon the authors – but perhaps they can act as inspiration for a more rigorous section.

Introduction

Major

• The authors state that "[..]has created new effective paradigms for analyzing high-dimensional data, e.g., in single-cell RNA- seq (scRNA-seq) research [19], the field of applying advanced computational methods to spatial transcriptomics data remains in its infancy [20]." However, the reference they cite here is from October 2017 and I would strongly argue that the field has developed much since. I do not contend that it’s a young field, but claiming that it’s in its infancy is slightly incorrect, given the plethora of new methods that are emerging and have already been presented. I would encourage the authors to reconsider this statement.

• The word "spot" is used quite ambiguously in the spatial transcriptomics sphere, where it represents the capture locations in the Visium platform, but in image-based methods it tends to indicate the fluorescent signal for each individual RNA molecule. Meanwhile, methods like Slide-seq{1,2} use the word "bead" to refer to the capture location. And many computational methods use "voxel" as a more general term.

Therefore, I would advice the authors to chose one of the following two alternatives:

1. Clearly define what is meant by a "spot" in this manuscript (i.e., introduce a stipulative definition)

2. Chose a more neutral word like "capture location".

Minor

The authors write "In multicellular organisms, arranged cells work collaboratively in intact tissues.". This is of course true, but cooperation between cells is not really exclusive to multicellular organisms, the authors but also occur in unicellular organisms, see Quorum sensing (https://en.wikipedia.org/wiki/Quorum_sensing). Implying that spatial techniques have an even broader application than only multicellular organisms.

• The statement "Protocols for scRNA-seq have achieved high-throughput gene expression profiling, but cellular spatial information is lost in scRNA-seq data (Fig. 1C).", is a bit contradictory since the authors also present methods for spatial reconstruction; meaning that at least some degree of spatial information is retained in the single cell/nuclei data.

Profiling of localized gene expression pattern

Major

• When speaking about SPARK the authors should consider mentioning that they also rely on Gaussian Processes in their model. They also launch the idea of a Generalized Linear Spatial Model (GLSM) which constitutes a large part of their "innovation" in terms of method development. One might also add that they use ten different kernels and combine the p-values by using the Cauchy combination rule. These are two aspects that sets the method apart from SpatialDE to some extent, and which I believe could be worth mentioning.

• The authors state that: "to generate the null distribution. In one application, Zhu et al. [31] utilize hidden Markov random fields (HMRF) to identify spatially variable genes. Zhu et al."

But the reference (Zhu et al.) reads: "Of note, SpatialDE does not identify spatial regions with distinct expression patterns. Similarly, trendsceek is also designed to detect spatial dependency. However, its application is limited to a single gene at a time. In contrast, our HMRF method can simultaneously detect the combinatorial pattern of all profiled genes. A unique aspect of our study was the integration of cell type and spatial domain annotations. "

My impression, although this could of course be incorrect, is that the HMRF approach that Zhu et al. use extracts spatial domains with similar expression profiles while also maintaining spatial cohesiveness. If my understanding is correct, this would not really put this method into the same category as SpatialDE or SPARK. But it’s more like a "spatially aware" clustering method.

Minor

• When the authors write "Specifically, the spatial component of the expression variation is modeled by the spatial covariance matrix based on the pairwise distances among locations[..]", for the unfamiliar reader it might be helpful to clarify that it is the spatial pairwise distance. For many people coming from the single cell world, the default "distance" is in gene expression space.

• I believe the authors might want to use "A Poisson link. . . " instead of "Poisson link. . . " in their text.

• The authors write: "A common theme of these aforementioned approaches [23, 24, 26, 27] is that they seek to identify spatially variable genes by testing whether gene expression levels in different spatial locations covary according to the spatial distances among them.", wouldn’t perhaps it be more fit to say that these methods test whether adding a spatial component to the covariance significantly increase its ability to explain the data?

• I believe sepal should be italicized. See: https://doi.org/10.1093/ bioinformatics/btab164.

Cellular deconvolution

Major

• The authors mention how using older methods designed for deconvolution of bulk RNA-seq data could theoretically be used for spatial transcriptomics data as well, which gives some "historical" context. Two other approaches that I would strongly recommend the users to cite are: – an NNMF approach suggested in: https://www.science.org/ doi/10.1126/science.aaw1219 – a marker gene based approach presented in: https://www.nature. com/articles/s41587-019-0392-8 As these represent some of the earlier solutions published for this type of task, and thus deserve some recognition.

• Although fairly recently published, and thus understandable why the authors might not have included it, I would consider it appropriate to also mention Tangram (https://www.nature.com/articles/ s41592-021-01264-7). This is a decomposition method that is compatible both with capture-based as well as image-based data, and use a fairly different approach (more optimization based) than the currently discussed methods.

• The authors also mainly speak about the spatial single cell mapping problem as something singularly used to decompose mixed observations, while truly it is also useful for label transfer and to avoid repeating annotations. One example of this is: https://www.nature.com/ articles/s41592-019-0631-4.

While not published yet, I believe Cell2location (see https://www. sanger.ac.uk/tool/cell2location/) very soon will be. To be relevant, the authors might want to consider mentioning it in their review (especially since they already cite some bioRxiv papers). This method has also been used in peer-reviewed publications: (https: //www.nature.com/articles/s41588-021-00972-2.pdf).

• The authors discuss of many of the methods specifically designed for spatial decomposition, but some methods like Seurat’s label transfer is very commonly used to "map single cell data as well", especially by less computationally inclined researchers who are more or less exclusively familiar with the Seurat suite. Given the widespread use of this method, it might be worthwhile mentioning in the main text. See [https://satijalab.org/seurat/archive/v3.2/spatial_ vignette.html](https://satijalab.org/seurat/archive/v3.2/spatial_%20vignette.html).

• The authors are of course free to choose their own headings, but I believe the task they refer to as cellular deconvolution is more commonly referred to as "spatial decomposition", "cell type mapping", or "single cell integration". See for example https://github.com/ open problems-bio/openproblems/issues/233 and https://www.nature. com/articles/s41592-020-01038-7.

• The authors write: "When a sequencing spot is larger than an individual cell, its measured gene expressions may be from a mixture of multiple cell types as the spot overlaps with multiple cells.". I would suggest to perhaps list a few examples of techniques which this is true for, as this would make it easier for the reader to put the material into context.

• This is a very minor detail, but if one should be fully correct it’s RCTD that adopts a similar approach to stereoscope and not the other way around. This is based on each method’s respective publication date.

• I also believe stereoscope should be italicized and without capitalization, see: <https://www.nature.com/articles/s42003-020-01247-y>

Gene Imputation

Major

• The method Tangram (see above) also provides means for imputation of spatial data leveraging single cell information. I would encourage the authors to also include this method in the given section. 3.4.2

Minor

• Similar to my comment in the "cell deconvolution" section, I think it might be helpful to give some examples of experimental techniques for which the statement: "Some spatial transcriptomics techniques have high capturing sensitivity, but they only sequence a small fraction of genes out of the entire transcriptome." hold true.

• The author writes: "gimVI [41], a neural network model, is proposed to integrate spatial transcriptomics data and scRNA-seq data for missing gene imputation. gimVI [41] is extended from scVI [42] and incorporates a conditional distribution to take the platform effect into consideration." This is fully true and accurate, but I also think one of the distinguishing factors with scVI and gimVI is how they also use neural networks to map some of the latent factors to parameter values in the model. This might be something that the authors want to mention. The authors of scVI also, in contrast to many other methods, provide a theoretical justification and conditions for their imputation.

Cell-Cell/Gene-Gene interactions

Major

• There is now a new (version 3) and updated version of CellPhoneDB presented in (https://www.nature.com/articles/s41588-021-00972-2. pdf), I believe this is more appropriate to cite than the older first version. 3.5.2

Minor

• This is very minor, but in the sentence: "[..]interactions, SVCA [65] utilizes Gaussian processes with additive covariance to model the variation of each gene expression." the authors might want to use the possessive form "gene’s" rather than "gene".

Conclusions and Perspectives

Major

• While not bad in any means, I personally consider (the conclusions and perspective section) this to be the "weakest" part of the manuscript. The promise of the section title and the paper abstract is to "aid" in future model development. However, I find the suggestions of problems that remains to be addressed or areas that are underexplored very vague. My current impression is that the authors mainly state that there are a lot of development is necessary but fail to give concrete examples of this, except for adapting trajectory inference to spatial data. 8 Some current challenges, that the authors could list, would be:

– 3D modeling and analysis of data

– The construction of common coordinate frameworks (CCFs) for spatial transcriptomics data. See, https://www.biorxiv.org/ content/10.1101/2021.11.11.468178v1.

– Spatiotemporal modeling. See, https://www.biorxiv.org/content/ 10.1101/757096v1

– Interaction analysis (based on the spatial data rather than single cell data). Between receptor and ligands, as well as different cell types. Also, important in the latter case, how we one can model multiway (more than two) interactions.

– Integration with other data modalities such as ATAC or protein signals. See for example, https://www.10xgenomics.com/ resources/datasets/invasive-ductal-carcinoma-stained-with-fluorescent-cd-3-antibody-1-standard-1-2-0

– There already exists a very extensive catalogue over the set of spatial experimental and computational method: https://pachterlab. github.io/LP_2021/. If the authors want to distinguish themselves from this, they should focus on providing more perspectives and provide "pointers" for the readers interested in developing new methods – but which may not have the proper background to identify the existing needs themselves.

Minor

• the authors could mention: https://www.pnas.org/content/pnas/ 116/39/19490.full.pdf, as an example of trajectory methods (Velocity) being adapted to spatial data (here MERFISH).

Major

There are some methods or set of methods that the authors have completely excluded from their survey, I will try to mention some of them here, and encourage the authors to maybe reconsider their inclusion.

• Methods to enhance the resolution of spatial transcriptomics data leveraging associated image data (often specific for Visium). There is one recent publication (Nature Biotechnology) on the matter where the idea of "super resolution spatial transcriptomics" is presented and applied to multiple tissue samples. This is a good example of how different data modalities can be joined together using methods of deep learning, and in my opinion deserves a place in this review. There was also a recent bioRxiv publication on the same topic:

– xFuse: <https://www.nature.com/articles/s41587-021-01075-3>

– HisToGene: [https://www.biorxiv.org/content/10.1101/2021. 11.28.470212v1.full](https://www.biorxiv.org/content/10.1101/2021.%2011.28.470212v1.full)

• There are also several methods that offer ways to infer copy number aberrations from spatial data, and which differ slightly from inferCNV by leveraging spatial proximity in their analysis to increase the robustness. One such example is STARCH: https://iopscience.iop.org/ article/10.1088/1478-3975/abbe99.

• Other methods also try to introduce a "spatial component" in their analysis workflow, for example MULTILAYER: https://www.cell. com/cell-systems/pdfExtended/S2405-4712(21)00151-4. Here they use an agglormerative clustering strategy to find spatial communities with similar expression profiles – but not using a HMRF approach.

• Another example of a "spatial" analysis trying to find higher-order interactions is scHOT (https://www.nature.com/articles/s41592-020-0885-x). While not exclusively devised for spatial data, its authors show its application (Figure 4) to in spatial transcriptomics data.

**Authors response**

Reviewer #1

In the manuscript entitled "Statistical and Machine Learning Methods for Spatially Resolved Transcriptomics Data Analysis", Zeng et al. mainly summarized the current statistical and machine learning methods for spatial transcriptomics data analysis with several different research areas, including gene expression spatial pattern identification, spatial transcriptomics data quality improvement (such as cell deconvolution and gene imputation), space reconstruction for scRNAseq data, and cell-cell/gene-gene interactions. Also, they provided a well-organized spatial transcriptomics data set and baseline methods list, which could be useful for further developing methods for spatial transcriptomics data analysis. Considering the rapid advancement and great power of spatial transcriptomics technology, summarizing this review about methods for spatial transcriptomics data analysis is meaningful and can help lots of researchers. However, this review mainly provided the brief introduction of different computational methods, lacking the systematic organization and summary of them. Therefore, I don't think the current version of this review meet the qualification for publication. My detailed comments are listed below and I hoped they would be helpful for the authors to further improve the manuscript.

*Response: We thank the Reviewer for this concise summary of our work. As shown below in our responses, we have carefully addressed the concerns raised and find that the constructive comments have helped to improve the manuscript in this revised version. The revised text that are incorporated in the updated manuscript are shown in blue below.*

1.1 The development background as well as interconnection of these types of methods covered in the review were not clearly described. For instance, though "cellular deconvolution" and "gene imputation" are both tools developed to improve data quality, they were specifical for two different types of spatial transcriptomics data. Also, space reconstruction is a unique analysis to predict spatial information for scRNA-seq data, which is quite different from other analysis of spatial transcriptomics data. For a logical review the authors would need to summarize all the methods according to the standard workflow of spatial transcriptomics data analysis.

*Response: We thank the Reviewer for the constructive suggestion. Now we have summarized the standard workflow of spatial transcriptomics data analysis in the Introduction section as suggested by the Reviewer. We have also outlined the development background and the interconnection between different methods in each topic-specific section of this manuscript. We also added an analysis workflow subfigure to Figure 1. The changes are reflected in the manuscript as:*

*“(Page 2) Introduction: A spatial transcriptomics data analysis workflow typically includes multiple phases (Fig. 1E). The first step is data preprocessing, which usually includes quality control, gene expression normalization, dimension reduction, and cell type annotation. One may further improve the data quality with spatial decomposition, gene imputation, and label transfer. Next, one could gain biological insights from the data through spatial clustering and localized gene expression pattern discovery, which could further facilitate\ the identification of spatially variable genes, inference of cell-cell/gene-gene interactions, as well as spatial trajectory analysis. Furthermore, spatial transcriptomics data can be utilized to help reconstruct the spatial locations of gene expressions in scRNA-seq data (Fig. 1E).”*

*(Page 3) Profiling of localized gene expression pattern: Genes differentially expressed with varying spatial patterns reflect biological functions. The identification of spatially variable genes is often the first downstream analysis in a standard workflow of analyzing spatial transcriptomics data. Early approaches of localized gene expression pattern identification include Trendsceek [24] and SpatialDE [25].*

*(Page 5) Spatial decomposition: When a capture location is larger than an individual cell, its measured gene expressions may be from a mixture of multiple cell types as the capture location overlaps with multiple cells. For example, the capture locations of Visium, a widely used microarray-based spatial transcriptomics technique, are 50 µm in diameter. This is often larger than a typical cell size (around 5-10 µm). Therefore, an important preprocessing step is to estimate the proportions of different cell types in each capture location using spatial decomposition algorithms, which is similar the concept of cellular deconvolution.*

*(Page 6) Gene imputation: Gene imputation is another major task to improve the quality of spatial transcriptomics data. Some spatial transcriptomics techniques have high capturing resolution, but they only sequence a small fraction of genes out of the entire transcriptome. For example, MERFISH may achieve single-cell resolution, but could only sequence around 1,000 genes [3]. Hence, to improve the quality of spatial transcriptomics data, one could impute the missing genes when performing data preprocessing.*

*(Page 8) Spatial location reconstruction for scRNA-seq data: The integration of scRNA-seq data and spatial transcriptomics data made spatial gene imputation possible. Likewise, spatial information derived from spatial transcriptomics could help reconstruct spatial information for scRNA-seq data.”*

Added in Figure 1:

1.2. For all the methods mentioned in this review, I think it would be clearer to list the principles, application scenarios, advantages and disadvantages of them into a table. Only simply describing how these methods work doesn't truly give useful guide to researchers.

*Response: We thank the Reviewer for this thoughtful suggestion. We have added a table of the principle algorithms, application scenarios, advantages, and disadvantages for the reviewed methods, as now reflected in Table 1.*

1.3 In the subsection "profiling of localized gene expression pattern", I only found the Trendsceek-liked and GCN-based methods in Fig.2. Why other machine learning methods such as sepal mentioned in the article were not showed in Fig.2? Additionally, SpaGCN and BayesSpace provided a spatial-clustering step, which is also an important research area in spatial transcriptomics data analysis. Therefore, it may be better to review other spatial clustering methods in a separate subsection.

*Response: We thank the Reviewer for this astute observation. Figure 2 mainly focused on two families of methods as they are the most widely used ones. Figures 2C and 2D illustrated the basic idea behind hypothesis-testing-based methods, such as Trendsceek, SpatialDE, SPARK, and SPARK-X, whereas Figure 2E demonstrated graph modeling, which is popular in spatial transcriptomics data analysis. We have now improved Figure 2 to indicate that we could also evaluate the degree of randomness in spatial expressions to find spatially variable genes (sepal’s approach). We have added a section to review spatial clustering methods in the manuscript as suggested by the Reviewer. We also moved BayesSpace to the spatial clustering section as BayesSpace aims to leverage information from spatial neighborhoods to enhance data resolution for spatial cluster identification. The changes are as reflected in the manuscript:*

*“(Page 4) The profiling of localized gene expression patterns is closely related to delineating spatially connected regions or clusters in a tissue based on expression data [34]. Indeed, spatial clustering is a critical step when performing exploratory analysis of spatial transcriptomics data, which may help reduce the data dimensionality and discover spatially variable genes. Standard clustering methods designed for scRNA-seq data were often based on highly variable genes, whereas spatial clustering requires us to take spatial information into account. To profile localized gene expression patterns, stLearn [35] first normalizes the expression data by smoothing the expression values in each capture location upon information aggregated from its neighbors and is weighted by the morphological similarity between capture locations. The capture locations are next clustered using standard algorithms such as k-means, and spatial information is used to refine the cluster results by merging subclusters from expression-driven clusters that are split across multiple spatially separated locations [35]. Inspired by contextual image classification methods, MULTILAYER [36] evaluates each gene’s differential expression level by comparing it to the average expression in the whole tissue and applies hierarchical agglomerative clustering to identify gene expression patterns. These patterns are represented by nodes in a graph, in which the edges are weighted by the similarities of gene patterns [36]. Tissue communities are next detected by applying Louvain clustering to the graph [36]. There are also examples that utilize Markov random fields (MRF) to incorporate spatial information when performing spatial clustering. Zhu et al. [33] utilize hidden Markov random fields (HMRF) to identify spatially variable domains. The authors first construct a neighborhood graph to represent the spatial relationship among the capture locations. Under the Markov assumption, cell states are only conditional on their immediate neighboring nodes. The authors leverage HMRF to decompose the graph into multiple components, and each component represents a spatially variable domain [33]. In another work, BayesSpace [37] employs a Bayesian approach and impose a prior to assign higher weights to spatial locations that are physically closer. However, the performance of BayesSpace [37] might be limited by its fixed smoothing parameter of the MRF. Moreover, it is not computationally scalable for high-throughput spatial transcriptomics data since its approach is fully Bayesian and hence computationally intensive [37]. To address these drawbacks, SC-MEB performs spatial clustering through an empirical Bayes approach capable of optimizing the smoothness parameter [38]. The gene expression at each capture location is assumed to be Gaussian given an unknown cluster label, and the prior of the hidden labels encourages spatial smoothness by penalizing the assignment of neighboring capture locations to different clusters [38]. SC-MEB estimates its parameters using an iterative-conditional-mode-based expectation-maximization method to boost its computational efficiency and scalability to high-throughput data [38]. Another strategy for spatial clustering is to perform graph-based clustering using both gene expression profiles and spatial features. STAGATE [39] is a graph attention auto-encoder framework capable of identifying spatial clusters. It first constructs a neighborhood graph of the capture locations, and prunes the graph based on the clustering of gene expressions [39]. The similarity between neighboring capture locations in the spatial graph is estimated by an attention layer, and clustering results on the inferred latent embeddings can then provide us with informative spatial domains [39]. In addition, as discussed in the previous section, SpaGCN is a GCN-based method capable of integrating gene expression, histology images, and spatial coordinates data [31]. In SpaGCN, spatial clusters are identified through clustering the output of the graph convolutional layer [31].”*

1.4 In the subsection "cellular deconvolution" and "gene imputation", methods with the same principles were supposed to be summarized together. For example, both spatialDWLS and SPOTlight deconvolute the spots of spatial transcriptomics data based on statistical regression, and describing them separately would cause confusion.

*Response: We agree with the Reviewer that separating the “cellular deconvolution" and "gene imputation” subsections could cause potential confusions, as they both belong to the data preprocessing step in the data analysis workflow. Now we have merged these two subsections and rephrased the introductions as necessary.*

1.5 In the subsection "conclusions and perspectives", due to most of methods have been reviewed in former subsection, summarizing them in detail again seemed not necessary. Moreover, the perspective of authors was not summarized clearly.

*Response: We thank the reviewer for the constructive comment. We have removed the redundant details in the “conclusions and perspectives” subsection. We also strengthened and better organized our perspectives, as reflected in the manuscript:*

*“(Page 12) Multiple exciting directions remain to be explored in the field. As new computational algorithms are being developed rapidly, the field will benefit from more systematic benchmark studies, like what has been done for scRNA-seq data analyses [99-101]. To facilitate this, we have summarized the datasets that have been used for tool development and benchmarking (Table S1) and the tools that have been used as baselines during method development (Table S2) in the papers reviewed. Comprehensive benchmark studies could aid potential users in prioritizing the methods that best fit their data and hypotheses. Computational algorithms have been developed to infer cell states and their developmental trajectories in scRNA-seq data [reviewed in [19]]. Even though most of the knowledge we learned from trajectory inference for scRNA-seq data is applicable to spatial transcriptomics data, it is necessary to adapt the algorithms so that they can utilize spatial information effectively, as has been done in [102]. A method called scHOT [103] is a computational approach designed to identify changes in higher-order interactions among genes in cells along a continuous trajectory or across space. This method has also been demonstrated to be effective in spatial transcriptomics data. In addition, incorporation of spatial location information has the potential to increase the sensitivity of cell-cell and gene-gene interaction studies, as interacting cells are more likely to be spatially adjacent. Indeed, with the rapid expansion of ligand-receptor interaction and cytokine secretion related knowledge [104], the integration of multiple data modalities might open new opportunities to study cell-cell and gene-gene interaction, especially the multiway interactions that involve multiple parties. Furthermore, with the fast development of sequencing technology, high-throughput platforms for spatial multi-omics are becoming available, for example, SM-Omics could capture both spatially resolved transcriptomes and proteomes [105], whereas SHARE-seq measures high-throughput ATAC and RNA expression simultaneously [106]. By employing these platforms, more levels of molecular information will be collected from the same tissue section. These data will provide a more holistic view of biological mechanisms and interactions but, at the same time, requires more sophisticated models with well-justified underlying assumptions for data analysis. Additionally, it is important to transfer the multimodal data to spatial space for visualization at single-cell resolution. For this purpose, further method development is needed to incorporate the spatial information into the multi-omics data. The construction of common coordinate frameworks (CCFs) is a computational approach to integrate data from various sources into a consistent reference and to construct maps of molecular and cellular organization at histological and anatomical scales. The concept of CCFs has been discussed in [107], and the method development has been tackled in [108]. CCFs have also been generated with 3D reference, which can be used to analyze, visualize, and integrate multimodal and multiscale datasets in 3D. In terms of 3D modeling, MERFISH has been extended to DNA imaging, which enables simultaneous imaging of the 3D organization of a tissue [109]. Computational approaches need to be developed to increase the efficiency of 3D data modeling and analysis. In addition, the analysis of spatial transcriptomics data from multiple tissue sections and timepoints has a potential to facilitate biological discovery, as has been done in [110]. Furthermore, neighboring cells in a tumor are likely to share similar copy number variations. Therefore, copy number inference on spatial transcriptomics data need to be tackled, such as in [111].”*

General summary

The authors set out to give a comprehensive account of the computational methods that exist for analysis of spatial transcriptomics data, as well as outlining some of the challenges that still remains to be addressed in the field. They list five broad main categories into which current techniques fall, being: • Profiling of localized gene expression pattern

• Cellular deconvolution

• Gene Imputation

• Spatial location reconstruction for scRNA-seq data

• Cell-cell/gene-gene interactions For each of these categories, they describe the objective of the associated methods and give examples of different strategies to achieve this. The work also contains a discussion of general pipelines for analysis of spatial trancsriptomics data as well as efforts to gather data sets.

General comments

Personally, I found the manuscript well structured and easy to read. The authors have written it in a short a concise format that makes it easy for the reader to get a "bird’s eye view" of the landscape of different computational methods. For the five topics they choose as overarching categories, they provide fairly extensive record of existing methods. However, there are some areas that if given additional attention, would elevate the quality of this manuscript even further. In general, I do feel as if some "historical" context is lacking with certain earlier methods not being mentioned or credited. While I fully understand that current methods are more relevant to the reader, I also believe that early contributors who "pioneered" the ideas should be mentioned; especially since these early methods often rely on simpler approaches that many readers may have contemplated themselves. The authors also mention that many of the techniques developed for analysis of single cell data could be and are applied to spatial data, but don’t really give examples of such analysis. These methods are not inherently spatial, but as they are commonly employed by researchers I also think mentioning them would be worthwhile. I would also try to discuss some additional flavors of methods in order to give a more comprehensive overview, potentially expanding the five categories (more on this later). Additionally, I have some comments regarding the statements and formulations used in the manuscript that I’ve taken the liberty to share with the authors, although I would not consider these as a requirement for my satisfaction, but rather just tips or input from someone with a different perspective. Finally, I would have preferred to see a slightly more explicit account of the future challenges, here I have some suggestions – which I by no means want to enforce upon the authors – but perhaps they can act as inspiration for a more rigorous section.

*Response: We thank the Reviewer for this concise summary of our work. As shown below in our detailed responses, we have carefully addressed the concerns raised and find that the constructive comments have helped to improve the manuscript in this revised version. The revised text that are incorporated in the updated manuscript are shown in blue below.*

2.1 Specific Comments.

This section contains more specific comments, which I’ve chosen to separate based on manuscript section and degree of importance (major vs. minor). I’ve also added a section called "Other" where there are comments that don’t pertain to a specific set of methods, mainly this include suggestions of additional methods that might benefit the work if they were included.

Introduction

Major.

The authors state that "[..] has created new effective paradigms for analyzing highdimensional data, e.g., in single-cell RNA- seq (scRNA-seq) research [19], the field of applying advanced computational methods to spatial transcriptomics data remains in its infancy [20]." However, the reference they cite here is from October 2017 and I would strongly argue that the field has developed much since. I do not contend that it’s a young field, but claiming that it’s in its infancy is slightly incorrect, given the plethora of new methods that are emerging and have already been presented. I would encourage the authors to reconsider this statement.

*Response: We thank the Reviewer for the comment. We have rewritten the statement to deliver the message more appropriately. As reflected in the manuscript:*

*“(Page 1) The recent development of computational approaches has created new effective paradigms for analyzing high-dimensional data, e.g., in single-cell RNA-seq (scRNA-seq) research [19]. Likewise, there has been much progress in the field of method development for spatial transcriptomics data analysis [20, 21].”*

2.2 The word "spot" is used quite ambiguously in the spatial transcriptomics sphere, where it represents the capture locations in the Visium platform, but in image-based methods it tends to indicate the fluorescent signal for each individual RNA molecule. Meanwhile, methods like Slideseq{1,2} use the word "bead" to refer to the capture location. And many computational methods use "voxel" as a more general term. Therefore, I would advice the authors to chose one of the following two alternatives:

1. Clearly define what is meant by a "spot" in this manuscript (i.e., introduce a stipulative definition) 2. Chose a more neutral word like "capture location"

*Response: Response: We thank the Reviewer for this suggestion. We agree with the Reviewer that the word spot could cause confusion. Therefore, we took the Reviewer’s suggestion and changed the word “spot” to the neutral word “capture location” across the manuscript.*

2.3 Minor The authors write "In multicellular organisms, arranged cells work collaboratively in intact tissues.". This is of course true, but cooperation between cells is not really exclusive to multicellular organisms, the authors but also occur in unicellular organisms, see Quorum sensing (https://en.wikipedia.org/wiki/Quorum_sensing). Implying that spatial techniques have an even broader application than only multicellular organisms.

*Response: We thank the Reviewer for pointing this out. We agree with the Reviewer that the cooperation between cells is not exclusive to multicellular organisms. We have rephrased the statement and added the statement about unicellular as well multicellular organisms in the manuscript. As reflected in the manuscript: “(Page 1) In unicellular and multicellular organisms, arranged cells work collaboratively in intact tissues”.*

2.4 The statement "Protocols for scRNA-seq have achieved high-throughput gene expression profiling, but cellular spatial information is lost in scRNA-seq data (Fig. 1C).", is a bit contradictory since the authors also present methods for spatial reconstruction; meaning that at least some degree of spatial information is retained in the single cell/nuclei data.

*Response: We thank the Reviewer for the comment. We agree with the Reviewer that spatial information is not completely lost in the single-cell data. As mentioned by the Reviewer, methods have been developed to derive hidden information in the single-cell data to reconstruct spatial information. Indeed, we meant to say that the capture location is not retained in the scRNA-seq data. We have rephrased the statement and the change is reflected in the manuscript*:

*“(Page 2) Protocols for scRNA-seq have achieved high-throughput gene expression profiling. Although information about cellular spatial location is lost during cell preparation (Fig. 1C), the lost information has a potential to be reconstructed by leveraging gene expression patterns of the cells.”*

2.5 Profiling of localized gene expression pattern Major When speaking about SPARK the authors should consider mentioning that they also rely on Gaussian Processes in their model. They also launch the idea of a Generalized Linear Spatial Model (GLSM) which constitutes a large part of their "innovation" in terms of method development. One might also add that they use ten different kernels and combine the p-values by using the Cauchy combination rule. These are two aspects that sets the method apart from SpatialDE to some extent, and which I believe could be worth mentioning.

*Response: We thank the Reviewer for pointing this out. We have added the unique aspects of SPARK, as reflected in the manuscript:*

*“(Page 3) Similar to SpatialDE [25], SPARK [27] is a generative model with a variety of kernels to detect genes with spatial variation. A Poisson link is used in the generalized linear model as the authors reasoned that spatial gene expression data is often present in the form of counts. In addition, SPARK [27] computes p-values using each of the kernels and utilizes the Cauchy combination rule [28] to combine the p-values. Using this approach, SPARK [27] produces well-calibrated p-values to control type I errors.”*

2.6 The authors state that: "to generate the null distribution. In one application, Zhu et al. [31] utilize hidden Markov random fields (HMRF) to identify spatially variable genes. Zhu et al." But the reference (Zhu et al.) reads: "Of note, SpatialDE does not identify spatial regions with distinct expression patterns. Similarly, trendsceek is also designed to detect spatial dependency. However, its application is limited to a single gene at a time. In contrast, our HMRF method can simultaneously detect the combinatorial pattern of all profiled genes. A unique aspect of our study was the integration of cell type and spatial domain annotations. " My impression, although this could of course be incorrect, is that the HMRF approach that Zhu et al. use extracts spatial domains with similar expression profiles while also maintaining spatial cohesiveness. If my understanding is correct, this would not really put this method into the same category as SpatialDE or SPARK. But it’s more like a "spatially aware" clustering method.

*Response: We agree with the Reviewer that the HMRF approach is mainly designed to identify spatial domains with similar expression profiles. Although it is not the main goal of the method, in one of the downstream applications, the authors performed differential expression analysis between the clusters to identify spatially variable genes. As suggested by the Reviewer, we have created a new subsection of spatial clustering methods and put this HMRF method in the new subsection. We also rewrote the method, as reflected in the manuscript:*

*“(Page 5) Zhu et al. [33] utilize hidden Markov random fields (HMRF) to identify spatially variable domains. The authors first construct a neighborhood graph to represent the spatial relationship among the capture locations. Under the Markov assumption, cell states are only conditional on their immediate neighboring nodes. The authors leverage HMRF to decompose the graph into multiple components, and each component represents a spatially variable domain [33].”*

2.7 Minor When the authors write "Specifically, the spatial component of the expression variation is modeled by the spatial covariance matrix based on the pairwise distances among locations[..]", for the unfamiliar reader it might be helpful to clarify that it is the spatial pairwise distance. For many people coming from the single cell world, the default "distance" is in gene expression space.

*Response: We thank the Reviewer for the comment. We have clarified that the distances were indeed spatial pairwise distance across the manuscript.*

2.8 I believe the authors might want to use "A Poisson link. . . " instead of "Poisson link. . . " in their text.

*Response: We thank the Reviewer for pointing this out. We have changed the phrase to “A Poisson link.*

2.9 The authors write: "A common theme of these aforementioned approaches [23, 24, 26, 27] is that they seek to identify spatially variable genes by testing whether gene expression levels in different spatial locations covary according to the spatial distances among them.", wouldn’t perhaps it be more fit to say that these methods test whether adding a spatial component to the covariance significantly increase its ability to explain the data?

*Response: We thank the Reviewer for the comment. We have rewritten the statement as suggested. The changes are reflected in the manuscript as:*

*“(Page 3) A common theme of these approaches [24, 25, 27, 29] is that they all test whether adding a spatial component to the covariance could significantly improve their ability to identify spatially variable genes.”*

2.10 I believe sepal should be italicized. See: https://doi.org/10.1093/ bioinformatics/btab164

*Response: We thank the Reviewer for the comment. we have italicized ‘sepal’ across the manuscript*

2.11 Cellular deconvolution

Major: The authors mention how using older methods designed for deconvolution of bulk RNA-seq data could theoretically be used for spatial transcriptomics data as well, which gives some "historical" context. Two other approaches that I would strongly recommend the users to cite are:

– an NNMF approach suggested in: https://www.science.org/ doi/10.1126/science.aaw1219

– a marker gene based approach presented in: https://www.nature. com/articles/s41587-019- 0392-8

As these represent some of the earlier solutions published for this type of task, and thus deserve some recognition.

*Response: We thank the Reviewer for the comment. As pointed out by the Reviewer, we have acknowledged that methods designed for bulk RNA-seq data deconvolution could theoretically be used for spatial transcriptomics data, adding historical context. We also have acknowledged that methods designed for bulk RNA-seq deconvolution have been systematically benchmarked in studies [71, 72]. We then used the tool DWLS as an example, which was originally designed for bulk RNA-seq data deconvolution, and was extended to study spatial data, namely spatialDWLS. As recommended by the Reviewer, we have cited the two studies recommended by the Reviewer. As reflected in the manuscript:*

*“(Page 6) To analyze Slide-seq data, Rodriques et al. [6] propose to utilize non-negative matrix factorization (NMF) to derive metagenes from the scRNA-seq data. With gene signatures representing each cell type inferred, the authors further leverage non-negative least square (NNLS) regression to map scRNA-seq cell types onto Slide-seq data.*

*(Page 6) With the derived cell type signatures, the probability of cell type mixtures in each capture location can be inferred through maximum likelihood estimation. Likewise, gene marker-based approaches have also been utilized to identify and map cell subpopulations across tissue regions [53].”*

2.12 Although fairly recently published, and thus understandable why the authors might not have included it, I would consider it appropriate to also mention Tangram (https://www.nature.com/articles/ s41592-021-01264-7). This is a decomposition method that is compatible both with capture-based as well as image-based data, and use a fairly different approach (more optimization based) than the currently discussed methods.

*Response: We thank the Reviewer for this suggestion. We have included Tangram in the review collection. As reflected in the manuscript:*

*“(Page 6) Other approaches have been proposed for spatial decomposition, for example, a recent method named Tangram [48]. Tangram [48] is an optimization-based approach to align scRNA-seq data onto different spatial transcriptomics data by enforcing the similarity between the two data types. It is worth noting that Tangram [48] is compatible with capture-based and image-based spatial transcriptomics data.”*

2.13 The authors also mainly speak about the spatial single cell mapping problem as something singularly used to decompose mixed observations, while truly it is also useful for label transfer and to avoid repeating annotations. One example of this is: https://www.nature.com/ articles/s41592-019-0631-4.

*Response: We thank the Reviewer for this comment. We have included the label transfer discussion in the manuscript, as reflected in the manuscript:*

*“(Page 8) The integration of scRNA-seq data and spatial transcriptomics data could improve data quality in many other ways. For example, Qian et al. developed a Bayesian model to leverage scRNA-seq data to estimate the probability of assigning each read to each cell and each cell to each class for transcriptomics data [66]. This is a typical use case of cell label transfer from scRNA-seq data to spatial transcriptomics data to assist cell type annotations. As part of the analysis workflow, cell type annotation is a major task to determine the cellular composition of complex tissues and organisms. The exponential growth in the number of cells and quality of scRNA-seq has prompted the adaption and development of computational approaches to transfer cell labels from scRNA-seq data to spatial transcriptomics data. Typically, the label transfer is performed in different ways. One could first learn gene markers or gene signatures representing cell types from the scRNA-seq data, and then computationally infer the cell types for spatial transcriptomics data by enrichment studies. Alternatively, one could integrate scRNA-seq and spatial transcriptomics data and compute their similarity to perform cell type annotation for spatial transcriptomics data, as in gimVI [54], Seurat [67], Tangram [48], and others.*

2.14 While not published yet, I believe Cell2location (see https://www. sanger.ac.uk/tool/cell2location/) very soon will be. To be relevant, the authors might want to consider mentioning it in their review (especially since they already cite some bioRxiv papers). This method has also been used in peer-reviewed publications: (<https://www.nature.com/articles/s41588-021-00972-2.pdf>)

*Response: We thank the Reviewer for this suggestion. We have included Cell2location in review collection. As reflected in the manuscript:*

*“(Page 6) A recent approach, Cell2location [49], is a hierarchical Bayesian model that maps the spatial distribution of cell types by leveraging information from scRNA-seq data. Cell2location [49] was systematically evaluated against other alternative methods, including stereoscope [45], Seurat [50], RCTD [44], NNLS (Autogenes) [51], and SPOTlight [52]. It was reported that Cell2location [49] outperformed these methods substantially in detecting the presence of cell types across locations.”*

2.15 The authors discuss of many of the methods specifically designed for spatial decomposition, but some methods like Seurat’s label transfer is very commonly used to "map single cell data as well", especially by less computationally inclined researchers who are more or less exclusively familiar with the Seurat suite. Given the widespread use of this method, it might be worthwhile mentioning in the main text. See [https://satijalab.org/seurat/archive/v3.2/spatial_ vignette.html](https://satijalab.org/seurat/archive/v3.2/spatial_%20vignette.html).

*Response: We thank the Reviewer for this comment. We agree with the Reviewer that single-cell mapping is also useful for label transfer. Indeed, cell label annotation is a major task for scRNA-seq analysis and has been systematically benchmarked [56]. Although computational approaches for automatic cell-type annotation are being developed, manual annotation still remains a major cell-type annotation method for scRNA-seq analysis. We included the label transfer as part of the analysis workflow as suggested by the Reviewer, reflected in the manuscript:*

*“(Page 7) The computational methods developed for spatial decomposition and gene imputation are largely dependent on the integration of scRNA-seq data and spatial transcriptomics data. Joint dimension reduction methods have been commonly used for this data integration purpose. A typical workflow for joint dimension reduction is to project multiple datasets to a common latent space based on feature similarities (Fig. 3B). With multiple datasets projected and clustered, complementary information from the other datasets could be used. For example, optimal transport algorithm [63] has been used in spatial transcriptomics data analyses [64, 65] as it could derive a probabilistic embedding to minimize the discrepancy between the shortest path lengths in expression data and spatial data. The integration of scRNA-seq data and spatial transcriptomics data could improve data quality in many other ways. For example, Qian et al. developed a Bayesian model to leverage scRNA-seq data to estimate the probability of assigning each read to each cell and each cell to each class for transcriptomics data [66]. This is a typical use case of cell label transfer from scRNA-seq data to spatial transcriptomics data to assist cell type annotations. As part of the analysis workflow, cell type annotation is a major task to determine the cellular composition of complex tissues and organisms. The exponential growth in the number of cells and quality of scRNA-seq has prompted the adaption and development of computational approaches to transfer cell labels from scRNA-seq data to spatial transcriptomics data. Typically, the label transfer is performed in different ways. One could first learn gene markers or gene signatures representing cell types from the scRNA-seq data, and then computationally infer the cell types for spatial transcriptomics data by enrichment studies. Alternatively, we could integrate scRNA-seq and spatial transcriptomics data and compute their similarity to perform cell type annotation for spatial transcriptomics data, as in gimVI [54], Seurat [67], Tangram [48], and others.”*

2.16 Minor The authors are of course free to choose their own headings, but I believe the task they refer to as cellular deconvolution is more commonly referred to as "spatial decomposition", "cell type mapping", or "single cell integration". See for example https://github.com/ openproblems-bio/openproblems/issues/233 and https://www.nature. com/articles/s41592-020-01038-7.

*Response: We thank the Reviewer for the comment. We have changed the heading to “spatial decomposition” as suggested by the Reviewer.*

2.17 The authors write: "When a sequencing spot is larger than an individual cell, its measured gene expressions may be from a mixture of multiple cell types as the spot overlaps with multiple cells.". I would suggest to perhaps list a few examples of techniques which this is true for, as this would make it easier for the reader to put the material into context.

*Response: We thank the Reviewer for the comment. As suggested by the Reviewer, now we use the VISIUM as an example. As reflected in the manuscript:*

*“*(Page 5) When a capture location is larger than an individual cell, its measured gene expressions may be from a mixture of multiple cell types as the capture location overlaps with multiple cells. For example, the capture locations of Visium, a widely used microarray-based spatial transcriptomics technique, are 50 µm in diameter. This is often larger than a typical cell size (around 5-10 µm). Therefore, an important preprocessing step is to estimate the proportions of different cell types in each capture location using spatial decomposition algorithms, which is similar to the concept of cellular deconvolution.”

2.18 This is a very minor detail, but if one should be fully correct it’s RCTD that adopts a similar approach to stereoscope and not the other way around. This is based on each method’s respective publication date.

*Response: We thank the Reviewer for pointing this out. We have rephrased the statement to avoid referencing the methods by timelines.*

2.19 I also believe stereoscope should be italicized and without capitalization, see: <https://www.nature.com/articles/s42003-020-01247-y>

*Response: We thank the Reviewer for pointing this out. We have italicized ‘stereoscope’ without capitalization.*

2.20 Gene Imputation

Major

The method Tangram (see above) also provides means for imputation of spatial data leveraging single cell information. I would encourage the authors to also include this method in the given section.

*Response: We thank the Reviewer for this comment. We have mentioned Tangram in the gene imputation section. As reflected in the manuscript:*

*“(Page 7) Some methods developed for spatial decomposition also have the gene imputation function, for example Tangram [48].”*

2.21 Minor Similar to my comment in the "cell deconvolution" section, I think it might be helpful to give some examples of experimental techniques for which the statement: "Some spatial transcriptomics techniques have high capturing sensitivity, but they only sequence a small fraction of genes out of the entire transcriptome." hold true.

*Response: We thank the Reviewer for the comment. Now we included the MERFISH example to hold our statement.*

*“(Page 6) Gene imputation is another major task to improve the quality of spatial transcriptomics data. Some spatial transcriptomics techniques have high capturing resolution, but they only sequence a small fraction of genes out of the entire transcriptome. For example, MERFISH may achieve single-cell resolution, but could only sequence around 1,000 genes [3]. Hence, to improve the quality of spatial transcriptomics data, one could impute the missing genes when performing data preprocessing.”*

2.22 The author writes: "gimVI [41], a neural network model, is proposed to integrate spatial transcriptomics data and scRNA-seq data for missing gene imputation. gimVI [41] is extended from scVI [42] and incorporates a conditional distribution to take the platform effect into consideration." This is fully true and accurate, but I also think one of the distinguishing factors with scVI and gimVI is how they also use neural networks to map some of the latent factors to parameter values in the model. This might be something that the authors want to mention. The authors of scVI also, in contrast to many other methods, provide a theoretical justification and conditions for their imputation.

*Response: We thank the Reviewer for the comment. As suggested by the Reviewer, now we highlighted the unique properties in scVI and gimVI. As reflected in the manuscript:*

*“(Page 7) For gene imputation purposes, gimVI [54], a neural network model, was proposed to integrate spatial transcriptomics data and scRNA-seq data for missing gene imputation. gimVI [54] is extended from scVI [55] and based on a hierarchical Bayesian model with conditional distributions specified by deep neural networks. Specifically, this latent representation is decoded by one additional nonlinear transformation to generate a posterior estimate of the distributional parameters of each gene in each cell. gimVI [54] and scVI [55] incorporate the conditional distribution to take platform effect into consideration. In terms of aggregating scRNA-seq data and spatial transcriptomics data, gimVI [54] and scVI [55] differ from other methods in their nonlinearity, as many other methods are dependent on linear models. Of note, gimVI [54] reasons that distributions of gene expression are platform-specific. It assumes a zeroinflated negative binomial (ZINB) distribution for scRNA-seq measurements, a Poisson distribution for single molecule fluorescence in situ hybridization (smFISH) [3] measurements, and a negative binomial distribution for spatially-resolved transcript amplicon readout mapping (starMAP) [56] measurements.”*

2.23 Cell-Cell/Gene-Gene interactions

Major There is now a new (version 3) and updated version of CellPhoneDB presented in (https://www.nature.com/articles/s41588-021-00972-2. pdf), I believe this is more appropriate to cite than the older first version.

*Response: We thank the Reviewer for the comment. As suggested by the Reviewer, now we cited the new version of CellphoneDB instead.*

2.24 Minor This is very minor, but in the sentence: "[..] interactions, SVCA [65] utilizes Gaussian processes with additive covariance to model the variation of each gene expression." the authors might want to use the possessive form "gene’s" rather than "gene".

*Response: We thank the Reviewer for pointing this out. We have revised the phrase accordingly.*

• While not bad in any means, I personally consider (the conclusions and perspective section) this to be the "weakest" part of the manuscript. The promise of the section title and the paper abstract is to "aid" in future model development. However, I find the suggestions of problems that remains to be addressed or areas that are underexplored very vague. My current impression is that the authors mainly state that there are a lot of development is necessary but fail to give concrete examples of this, except for adapting trajectory inference to spatial data. 8 Some current challenges, that the authors could list, would be:

– 3D modeling and analysis of data

– The construction of common coordinate frameworks (CCFs) for spatial transcriptomics data. See, https://www.biorxiv.org/ content/10.1101/2021.11.11.468178v1.

– Spatiotemporal modeling. See, https://www.biorxiv.org/content/ 10.1101/757096v1

– Interaction analysis (based on the spatial data rather than single cell data). Between receptor and ligands, as well as different cell types. Also, important in the latter case, how we one can model multiway (more than two) interactions.

– Integration with other data modalities such as ATAC or protein signals. See for example, https://www.10xgenomics.com/ resources/datasets/invasive-ductal-carcinoma-stained-with-fluorescent-cd-3-antibody-1-standard-1-2-0

– There already exists a very extensive catalogue over the set of spatial experimental and computational method: https://pachterlab. github.io/LP_2021/. If the authors want to distinguish themselves from this, they should focus on providing more perspectives and provide "pointers" for the readers interested in developing new methods – but which may not have the proper background to identify the existing needs themselves.

*Response: We thank the Reviewer for the constructive comment. We have added more content and discussions in this subsection. Specifically, we have reorganized this subsection and added more future work discussions, as suggested by the Reviewer. The discussed topics include benchmarking, trajectory inference, cell-cell/gene-gene interactions, data modality integration, and other multiple topics. We thank the Reviewer for suggesting us the topics to be discussed in detail. The changes are now reflected in the manuscript as:*

*“(Page 12) Multiple exciting directions remain to be explored in the field. As new computational algorithms are being developed rapidly, the field will benefit from more systematic benchmark studies, like what has been done for scRNA-seq data analyses [99-101]. To facilitate systematic benchmark studies, we have summarized the datasets that have been used for tool development and benchmarking (Table S1) and the tools that have been used as baselines during method development (Table S2) in the papers reviewed. Comprehensive benchmark studies could aid potential users in prioritizing the methods that best fit their data and hypotheses. Computational algorithms have been developed to infer cell states and their developmental trajectories in scRNA-seq data [reviewed in [19]]. Even though most of the knowledge we learned from trajectory inference for scRNA-seq data is applicable to spatial transcriptomics data, it is necessary to adapt the algorithms so that they can utilize spatial information effectively, as has been done in [102]. A method called scHOT [103] is a computational approach designed to identify changes in higherorder interactions among genes in cells along a continuous trajectory or across space. This method has also been demonstrated to be effective in spatial transcriptomics data. In addition, incorporation of spatial location information has the potential to increase the sensitivity of cell-cell and gene-gene interaction studies, as interacting cells are more likely to be spatially adjacent. Indeed, with the rapid expansion of ligand-receptor interaction and cytokine secretion related knowledge [104], the integration of multiple data modalities might open new opportunities to study cell-cell and gene-gene interaction, especially the multiway interactions that involve multiple parties. Furthermore, with the fast development of sequencing technology, high-throughput platforms for spatial multi-omics are becoming available, for example, SMOmics could capture both spatially resolved transcriptomes and proteomes [105], whereas SHARE-seq measures high-throughput ATAC and RNA expression simultaneously [106]. By employing these platforms, more levels of molecular information will be collected from the same tissue section. These data will provide a more holistic view of biological mechanisms and interactions but, at the same time, requires more sophisticated models with well-justified underlying assumptions for data analysis. Additionally, it is important to transfer the multimodal data to spatial space for visualization at single-cell resolution. For this purpose, further method development is needed to incorporate the spatial information into the multi-omics data. The construction of common coordinate frameworks (CCFs) is a computational approach to integrate data from various sources into a consistent reference and to construct maps of molecular and cellular organization at histological and anatomical scales. The concept of CCFs has been discussed in [107], and the method development has been tackled in [108]. CCFs have also been generated with 3D reference, which can be used to analyze, visualize, and integrate multimodal and multiscale datasets in 3D. In terms of 3D modeling, MERFISH has been extended to DNA imaging, which enables simultaneous imaging of the 3D organization of a tissue [109]. Computational approaches need to be developed to increase the efficiency of 3D data modeling and analysis. In addition, the analysis of spatial transcriptomics data from multiple tissue sections and timepoints has a potential to facilitate biological discovery, as has been done in [110]. Furthermore, neighboring cells in a tumor are likely to share similar copy number variations. Therefore, copy number inference on spatial transcriptomics data need to be tackled, such as in [111].”*

2.26 Minor the authors could mention: https://www.pnas.org/content/pnas/ 116/39/19490.full.pdf, as an example of trajectory methods (Velocity) being adapted to spatial data (here MERFISH).

*Response: We thank the Reviewer for the comment. As suggested by the Reviewer, we have included this method as an example in the manuscript.*

*“(Page 12) Even though most of the knowledge we learned from trajectory inference for scRNA-seq data is applicable to spatial transcriptomics data, it is necessary to adapt the algorithms so that they can utilize spatial information effectively, as has been done in [102].”*

2.27 Other Major There are some methods or set of methods that the authors have completely excluded from their survey, I will try to mention some of them here, and encourage the authors to maybe reconsider their inclusion.

• Methods to enhance the resolution of spatial transcriptomics data leveraging associated image data (often specific for Visium). There is one recent publication (Nature Biotechnology) on the matter where the idea of "super resolution spatial transcriptomics" is presented and applied to multiple tissue samples. This is a good example of how different data modalities can be joined together using methods of deep learning, and in my opinion deserves a place in this review. There was also a recent bioRxiv publication on the same topic: – xFuse: https://www.nature.com/articles/s41587-021-01075-3 – HisToGene: [https://www.biorxiv.org/content/10.1101/2021. 11.28.470212v1.full](https://www.biorxiv.org/content/10.1101/2021.%2011.28.470212v1.full)

• There are also several methods that offer ways to infer copy number aberrations from spatial data, and which differ slightly from inferCNV by leveraging spatial proximity in their analysis to increase the robustness. One such example is STARCH: https://iopscience.iop.org/ article/10.1088/1478-3975/abbe99.

• Other methods also try to introduce a "spatial component" in their analysis workflow, for example MULTILAYER: https://www.cell. com/cell-systems/pdfExtended/S2405-4712(21)00151-4. Here they use an agglormerative clustering strategy to find spatial communities with similar expression profiles – but not using a HMRF approach.

• Another example of a "spatial" analysis truing to find higher-order interactions is scHOT (https://www.nature.com/articles/s41592-020-0885-x). While not exclusively devised for spatial data, its authors show its application (Figure 4) to in spatial transcriptomics data.

*Response: We thank the Reviewer for the comment. We have addressed the comment by discussing the various topics in different sections, as reflected in the manuscript:*

*“(Page 8) To further improve the quality of spatial transcriptomics data, methods have been developed to leverage other data types in addition to scRNA-seq data. For example, xFuse [68] is a deep generative model that integrates in situ RNA capturing data with histology image data to infer transcriptome-wide expression maps. The quantification of gene expression both within and between the original capture locations enhances the resolution of spatial transcriptomics data. HistoGene [69] is another deep learning model to leverage information learned from spatial transcriptomics data to predict gene expression for tissue sections where only histology images are available. HistoGene [69] outperforms other approaches that were designed to predict gene expression profiles from whole-slide images, including a multilayer perceptron-based method HE2RNA [70] and a supervised convolutional neural network-based approach ST-Net [71].*

*(Page 12) Furthermore, neighboring cells in a tumor are likely to share similar copy number variations. Therefore, copy number inference on spatial transcriptomics data need to be tackled, such as in [111].*

*(Page 4) Inspired by contextual image classification methods, MULTILAYER [36] evaluates the differential expression of genes relative to the average expression in the whole tissue and applies hierarchical agglomerative clustering to identify gene expression patterns. These patterns are represented by nodes in a graph, in which the edges are weighted by the similarities among the gene patterns [36]. Tissue communities are next detected by applying Louvain clustering to the graph [36].*

*(Page 12) Even though most of the knowledge we learned from trajectory inference for scRNA-seq data is applicable to spatial transcriptomics data, it is necessary to adapt the algorithms so that they can utilize spatial information effectively, as has been done in [102]. A method called scHOT [103] is a computational approach designed to identify changes in higher-order interactions among genes in cells along a continuous trajectory or across space. This method has also been demonstrated to be effective in spatial transcriptomics data”*

**Second round reviews**

**Reviewer 1:**

In their revised manuscript, Zeng et al have tried to address the issues that I brought up. The major revisions include:
.summarizing the standard workflow of spatial transcriptomics data analysis in the "introduction" subsection
.providing a summarized table of the principle algorithms, application scenarios, advantages, and disadvantages for different analysis methods
.adding a separate subsection of "spatial clustering"
.re-organizing the "conclusions and perspectives" subsection
These revisions truly elevate the quality of this manuscript even further, and help to fit the topic of spatial transcriptomics data analysis methods better. However, I still have some remaining concerns that I would like the authors to address.

Introduction
1.The author state that "The first step is data preprocessing, which usually includes […]". It's worth noting that the data preprocessing of imaging-based spatial transcriptomics contains a unique cell segmentation step, and a lot of methods have been developed, such as the cell segmentation method used in MERFISH (PMID:30385464), as well as several advanced methods published recently, such as Baysor (PMID:34650268), JSTA (PMID:34057817), and so on.
2.The statement "Next, we discuss computational methods that learn patterns from spatial transcriptomics data to help reconstruct spatial information for scRNA-seq data" is kind of inappropriate. To my knowledge, some methods such as novoSpaRc ((PMID:31748748) and CSOmap (32541867) don't need spatial transcriptomics data reference.

Profiling of localized gene expression pattern
3.Only adding the statement "assess the degree of randomness/structure" in Figure 2 is kind of confusing. It would be better to mention it when reviewing the "speal" method.

Spatial clustering
4.In my own opinion, in the statement "Standard clustering methods designed for scRNA-seq data were often based on highly variable genes", using "gene expression level" rather than "highly variable genes" would be more appropriate.

Spatial decomposition and gene imputation
5.Before reviewing different methods, summarizing the different application scenarios of "spatial decomposition" and "gene interpolation" methods briefly would be clearer to readers. Although both "spatial decomposition" and "gene interpolation" belong to the data preprocessing step, they address different problems. The "spatial decomposition" methods are designed for spatially resolved transcriptomics data that cannot reach the single-cell resolution, while "gene interpolation" methods are used for imaging-based spatial resolved transcriptomics data that measure a limited number of target genes.
6.It would be better to categorize and review different spatial decomposition methods based on their principle algorithms. For example, both spatialDWLS, NNLS, and SPOTlight use the nonnegative least square regression or its variants to deconvolute the spots of spatially resolved transcriptomics data, and a major difference between them is the strategy of constructing the gene signature matrix. So, from my perspective, reviewing them together would be more appropriate.
7.Panel C is missing in Figure 3.
8.The author state that "[…], the application of graph-based methods may improve the imputation of spatial genes". Have any such analysis methods been published so far? If so, maybe you could list some examples.

Conclusion and perspectives
9.I agree well with these remaining explored directions proposed by the author in the subsection "conclusion and perspectives", and some areas could be extended:
.Similar to identifying spatially variable genes, finding spatially variable cells (those annotated as the same cluster in scRNA-seq data but actually locate in different spaces and possibly perform different functions) is also important. For example, in recent research, cell2location has detected some regional astrocyte subtypes.
.There have been lots of multiple modalities integration methods published for single-cell ATAC and RNA data, but the integration of single-cell ATAC data with spatial RNA data is still relatively rare (Tangram has made some attempts), and it may be a direction for subsequent research.
.The construction of common coordinate frameworks (CCFs) is important but difficult. Due to the location, shape, size, and technology of spatial slices being different, how to align and integrate the spatial slices in different conditions still needs to be addressed. Comparing different slices with their relative positions may be a trade-off.

**Reviewer 2:**

General comment

The authors have done an excellent revision job and, as a result, the quality of this manuscript has significantly increased. It now presents as a useful resource for people with an interest in the field and provides a concise overview of the existing analysis methods. I have some fairly minor comments, which I’d like the authors to consider attending to; these mainly consist of technical details and small errors. If the authors act upon these comments, I see this manuscript as more than fit for publication and would encourage the editors to publish it as soon as possible in order to keep the material relevant and up-to-date.

Immediate replies to the authors’ response: Reply to comment 2.6 - The authors have added the following text to their manuscript: “[..] The authors first construct a neighborhood graph to represent the spatial relationship among the capture locations. Under the Markov assumption, cell states are only conditional on their immediate neighboring nodes.”

While I strongly support the addition of this paragraph to the manuscript, I would perhaps reconsider the sentence highlighted in bold. In the HMRF model employed by Zhu et al., the cell states indeed depend on the label of their immediate neighbor nodes, but the potential function also includes a term that compares the expression of a cell with the different clusters. In essence, the HMRF model forces the clusters to be coherent both in physical and gene expression space.

Reply to comment 2.21 - It’s great to see that the authors have tried to include an example of a method with high capture sensitivity. However, maybe the authors should make sure that their use of the term “capture sensitivity” aligns well with previous literature, where, I believe, this term is usually used to describe how many of the available transcripts that a method can register, see https://pachterlab.github.io/LP_2021/current-techs.html. The aspect which the authors refer to as sensitivity in their manuscript, to me, seems more like a question about resolution.

Also, the MERFISH technique has been shown to be able to register about 10000 genes, which is 10x more than the number given by the authors, see https://www.pnas.org/content/116/39/19490. However, I do believe ~1000 genes is the more common number of genes queried with MERFISH.

New comments:

● Minor: The authors write “We also discuss strategies for improving the data quality, including spatial decomposition and gene imputation (Fig. 1B).” I would perhaps choose another phrase than “improving the data quality”, both the factor decomposition and gene imputation are conditioned on the observed data and will not actually improve the quality of the data, however, it does increase the richness of the data or increase number of features associated with the data. But a factor decomposition or gene imputation will not have any effect on the quality of the experimentally derived data.

● Major: The authors claim that: “The identification of spatially variable genes is often the first downstream analysis in a standard workflow of analyzing spatial transcriptomics data.” I’ve reviewed and written many papers encompassing analysis spatial transcriptomics data, and would perhaps contest this statement. It is true that gene selection is usually one of the first steps in downstream analysis, but this selection is rarely based on spatial variability. Rather, other metrics and methods are used to extract a set of informative genes to work with. See the Methos sections of:

○ https://www.nature.com/articles/s41467-021-26271-2

https://www.nature.com/articles/s41467-022-28497-0#Sec16 <https://www.nature.com/articles/s41588-021-00972-2#Sec10>

Whether the current approach is the best procedure or not, I will let others debate, but using spatially variable genes is definitely not - yet - the most common approach.

● Minor: The authors now mention the updated SPARK-X method, perhaps, for comprehensiveness, they also want to add some information about the updated SpatialDE2 method, see <https://www.biorxiv.org/content/10.1101/2021.10.27.466045v2>.

● Minor: In their description of BayesSpace, the authors write: “Moreover, it is not computationally scalable for high-throughput spatial transcriptomics data since its approach is fully Bayesian and hence computationally intensive [37].” Is it really the Bayesian aspect that makes the model computationally expensive? Bayesian models can scale well if the integrals are tractable or variational inference is used, I’d rather say that it’s the use of MCMC part that makes BayesSpace scale poorly.

● Major: the authors write: “For example, the capture locations of Visium, a widely used microarray-based spatial transcriptomics technique, are 50 µm in diameter.” In fact, a Visium spot is 55µm in diameter, not 50. For reference, see https://kb.10xgenomics.com/hc/en-us/articles/360035487572-What-is-the-spatial-resolution-and-configu ration-of-the-capture-area-of-the-Visium-Gene-Expression-Slide-.

● Major: The authors write: “Specifically, stereoscope [45] assumes that spatial gene counts follow the negative binomial distribution, whereas RCTD [44] assumes a Poisson distribution.” The statement about stereoscope using a negative binomial distribution is true, but maybe the authors want to revise their statement about RCTD. The negative binomial arise from modeling the gene expression with a hierarchical gamma-Poisson model; the Poisson part is used to model the capture of transcripts from the common pool of transcripts (e.g., in a cell or spot) while the gamma distribution is put as a prior on the Poisson mean to account for biological variability. In RCTD, Cable et al. places a lognormal as a prior on their Poisson distribution’s mean, effectively producing a Poisson-lognormal distribution for the gene expression. I refer to the supplementary of the original RCTD manuscript for more details.

● Minor: the sentence “It is also worth noting that Bageritz et al. [81] have identified 824 genes with spatially restricted expression patterns, which can potentially be used as a spatial reference map.” Seems to be taken completely out of context to me? Are these 824 common to all spatial tissues, or does it pertain to specific organisms/tissue?

● Major: I would really encourage the authors to mention the model described in https://www.biorxiv.org/content/10.1101/2021.07.11.451750v1 in their Cell-Cell/Gene-Gene interactions. This method shows some really innovative ideas and is likely to be the first of many using these concepts.

● Minor: The authors state that “As the field is working towards transcriptome-wide sequencing, spatial transcriptomics data quality is[..]” The Spatial Transcriptomics paper by Ståhl et al. (2016) presents a method that is transcriptome-wide, and the same is true for the 2019 Slide-seqV1 manuscript. I’m not sure whether “working towards” really gives an accurate description of the current status of the field, as this objective has since long been achieved.

● Minor: In addition to the MERFISH platform, the Slide-seq platform has also been extended to DNA assays (see https://www.biorxiv.org/content/10.1101/2021.06.06.447244v1). Also, the Spatial CUT&Tag platform has been published <https://www.science.org/doi/10.1126/science.abg7216>.

● Major: The authors now speak about interrogating the CNV landscape of spatial tissues, this has already been done in a systematic manor: <https://www.biorxiv.org/content/10.1101/2021.07.12.452018v1>.
